# High-yielding and scalable synthesis of furfural acetals using protonated κ-carrageenan as a biorenewable acid catalyst

**DOI:** 10.1039/d5ra10090a

**Published:** 2026-01-22

**Authors:** Rachitha S. Natraj, Saikat Dutta

**Affiliations:** a Department of Chemistry, National Institute of Technology Karnataka (NITK) Surathkal Mangalore 575025 Karnataka India sdutta@nitk.edu.in

## Abstract

The acetals of carbohydrate-derived furfurals have potential applications as fuel oxygenates and chemical intermediates. This work reports the use of protonated κ-carrageenan (kCH) as a marine biomass-derived renewable acid catalyst for the acetalization of furfural and 5-methylfurfural with ethylene glycol, 1,2-propanediol, and 1,3-propanediol. Since these glycols have promising catalytic production routes from biomass, the corresponding acetals are biorenewable to their entirety. Cyclohexane was used as the water removal agent by azeotropic distillation in a Dean–Stark apparatus. The isolated yields of the acetals were excellent (>90%), and the 2-(furan-2-yl)-1,3-dioxolane was conveniently prepared on a 10 g scale. The broad substrate scope of the catalyst was demonstrated by the successful preparation of acetals from benzaldehyde in excellent isolated yields. The kCH catalyst was conveniently recovered after the reaction and reused without catastrophic loss in catalytic activity. The effect of the extent of proton exchange on the activity, thermal stability, and recyclability of the kCH catalyst was also explored.

## Abstract 2

The acetals of carbohydrate-derived furfurals have potential applications as fuel oxygenates and chemical intermediates.

## Graphical abstract

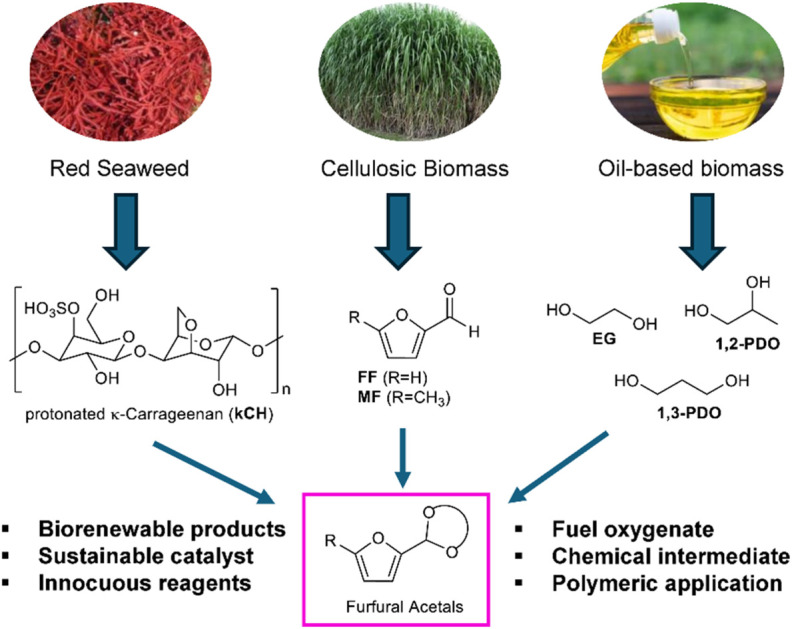

## 1 Introduction

The organic chemical manufacturing industries are undergoing a transition to integrate biomass as a renewable carbon-based feedstock alongside traditional fossil-based exhaustible resources (*e.g.*, petroleum).^1^ Using renewable biomass as the starting material for liquid transportation fuels, organic chemicals, and synthetic polymers has clear societal, economic, and environmental advantages.^2^ Among the various biomass value-addition technologies, the catalytic pathway has received particular attention. The catalytic transformation of biomolecules into targeted organic molecules is fast, scalable, highly selective, energy-efficient, and reagent-economical.^3^ Moreover, the processes can be conveniently incorporated into the existing petrorefinery infrastructure. The structurally diverse polymeric biomolecules can be transformed into functionalized organic molecules in a two-step approach. The biomolecules are initially transformed into a handful of functionalized organic molecules, known as platform chemicals.^4^ Synthetic upgrading of the platform molecules in the second step leads to molecules of desired structural characteristics and properties.^5^

Carbohydrates constitute the major fraction of most biomasses, and they have received much attention as feedstock for synthesizing biofuels and biochemicals.^6,7^ Cellulose and hemicellulose are abundant carbohydrates in the second-generation biomass, whereas abundant carbohydrates in the third-generation marine biomass include carrageenans, ulvans, and alginates.^8^ Furfural (FF) and 5-(hydroxymethyl)furfural (HMF) are at the forefront of biorefinery research as carbohydrate-derived platform chemicals.^9,10^FF and HMF have been known in the literature for more than a century, and they have received renewed attention over the past three decades due to the commercial value of many of their derivatives.^11^FF is produced by the acid-catalyzed dehydration of pentose sugars, such as xylose and arabinose, in the hemicellulose fraction of lignocellulosic biomass.^12^HMF is produced by the dehydration of hexose sugars (*e.g.*, glucose, fructose) and polymeric carbohydrates containing hexoses (*e.g.*, starch, cellulose).^13^HMF has also been produced from marine carbohydrates, such as carrageenans.^14^FF and HMF retain all the biogenic carbon atoms of the parent sugar molecule, as well as some reactive functionalities. Moreover, the catalytic dehydration process produces water as the sole innocuous byproduct. The functionalities present in FF and HMF have been exploited for their synthetic value addition to virtually all classes of organic chemicals of industrial value.^15^ 5-Methylfurfural (MF) is an important furanic platform chemical produced from hexose sugars and polymeric carbohydrates.^16^ The catalytic hydrogenation reaction can produce MF by partially reducing isolated HMF.^17^MF can be produced from hexoses in a one-pot process without isolating the HMF as an intermediate.^18^MF has also been produced by reducing the hydrophobic analogs of HMF, such as 5-(chloromethyl)furfural.^19^MF can also directly be produced by the acid-catalyzed dehydration of specific sugars (*e.g.*, l-rhamnose).^20,21^

Acetalization is a prominent reaction for aldehyde functionality in FF and MF. The acetals of FF and MF have direct applications as potential fuel oxygenates.^22^ Moreover, the acetals can act as chemical intermediates for downstream synthetic value addition pathways, as well as polymeric applications.^23^ Acetalization of the aldehyde group in FF or MF increases the electron density on the furan ring, allowing it to participate in specific transformations, such as the [4 + 2] Diels–Alder reaction, to synthesize polyfunctionalized bioaromatics.^24^

**Scheme 1:**
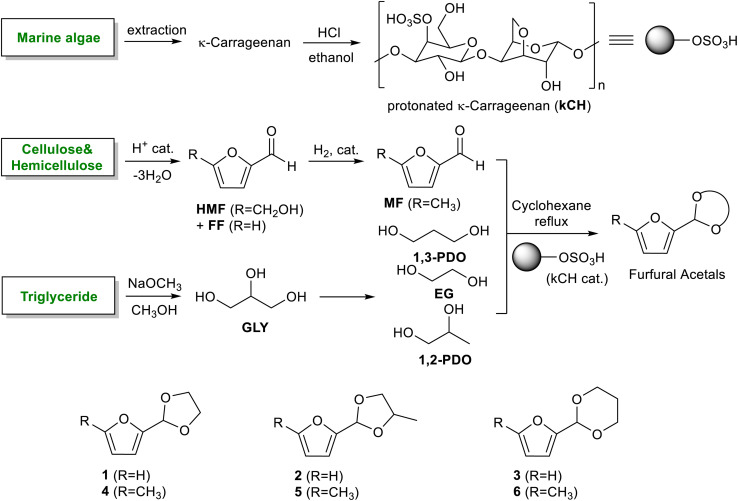
Acetalization of carbohydrate-derived furfurals and glycols using protonated κ-carrageenan as the biomass-derived renewable heterogeneous catalyst.

The cyclic acetals of FF and MF have received particular interest, as they exhibit significantly better hydrolytic and storage stability than their acyclic counterparts. Ethylene glycol (EG), 1,2-propanediol (1,2-PDO), and 1,3-propanediol (1,3-PDO) are routinely used for synthesizing cyclic acetals of FF and MF.^25,26^ These compounds have established renewable production routes. For example, EG can be produced by oxidizing ethylene, which is derived from the dehydration of bioethanol.^27^ Alternatively, the retro-Diels–Alder reaction of glucose under hydrothermal conditions in the presence of a suitable Lewis acid catalyst can lead to glycolaldehyde, which, on hydrogenation, forms EG.^28^ The selective catalytic reduction of triglyceride-derived glycerol (GLY) can produce 1,2-PDO and 1,3-PDO.^29,30^EG can be produced GLY through the C–C bond-cleavage reaction under hydrothermal conditions.^31^ Therefore, the acetals formed by FF or MF with these diols and triols are biorenewable to their entirety (Scheme 1).

Numerous homogeneous and heterogeneous acid catalysts have been developed for the acetalization reaction of aromatic aldehydes, including furfurals.^32–34^ There is still enormous scope for developing new catalysts that are robust, inexpensive, recyclable, eco-friendly, possessing a broad substrate scope, working under moderate conditions, and affording high selectivity and yield of catalysts. Catalysts sourced from biomass have garnered attention due to their renewable and environmentally friendly characteristics.^35,36^ The use of biomass-derived catalysts for synthesizing these renewable molecules would certainly expand the scope of introducing carbon atoms of biogenic origin in synthesizing value-added organic chemicals, especially in the context of a carbohydrate-centric biorefinery.^37^ Polymeric carbohydrates, functionalized with appropriate functionalities, have been used as catalysts for various organic transformations.^38^ We had reported using cellulose sulfuric acid (CSA), produced by sulfonating semicrystalline cellulose, as the heterogeneous acid catalyst for the ketalization of levulinic esters, which are also carbohydrate-derived platform chemicals.^39^ κ-Carrageenan (kC) is a naturally occurring sulfated carbohydrate that is found in red seaweed (*e.g.*, *Kappaphycus alvarezii*), which can be protonated by treating with a strong mineral acid.^14^

In this work, we report the use of protonated κ-carrageenan (kCH) as a novel heterogeneous acid catalyst for synthesizing the cyclic acetals of FF and MF using EG, 1,2-PDO, and 1,3-PDO. The reaction was performed in a Dean–Stark apparatus using cyclohexane as the solvent and azeotropic water removal. The effects of various reaction parameters, including the molar ratio of reagents and catalyst loading, on the selectivity and isolated yields of acetals were investigated. The kCH catalysts (both fresh and recycled) were characterized extensively using various analytical techniques, including FTIR, PXRD, FESEM, and TGA.

## 2 Materials, instruments, and experimental procedures

### 2.1 Materials and methods

Furfural (99%) was purchased from Spectrochem Pvt. Ltd. 5-Methylfurfural (MF, 99%) and Amberlyst-15 were purchased from Sigma. Ethyl acetate (99%) was purchased from Finar Limited. Cyclohexane (99.5%) was purchased from Merck. Ethylene glycol (99%), 1,3-propanediol (98%), 1,2-propanediol (99%), sodium sulfate (anhydrous, 99%), and HCl (35%, aq.) were purchased from Loba Chemie Pvt. Ltd. Furfural was distilled and refrigerated in an airtight, amber-colored glass container. All other chemicals are used as received without further purification. Thin-layer chromatography (TLC) plates and silica gel pre-coated on aluminum sheets were purchased from Merck (TLC Silica Gel 60, F254).

### 2.2 Instruments and characterization of the catalyst

The synthesized acetal products were characterized by spectroscopic techniques. The Fourier transform infrared (FTIR) spectra of the organic compounds were collected using a Bruker Alpha II FTIR 4000 instrument equipped with the attenuated total reflectance (ATR) technique. FTIR spectra of the materials (kC, fresh kCH-1, recycled kCH-1, and fresh kCH-2) were collected using the same instrument by pelletizing them with anhydrous KBr. The crystallinity of the samples was analyzed from Bragg's diffraction angles (2*θ*) using an Empyrean 3rd Gen powder X-ray diffractometer (PXRD) from Malvern PANalytical, Netherlands. Field-emission scanning electron microscopy (FE-SEM) images were collected using Thermo Fisher, FEI QUANTA 250 FEG. Samples were prepared by spin-coating the solutions onto a silicon substrate and dried under vacuum at room temperature. Samples were sputtered with gold for FE-SEM measurements. Elemental mapping of carbon, oxygen, potassium, and sulfur was done using a Thermo Fisher, FEI QUANTA 250 FEG (EDS). The Brunauer–Emmett–Teller (BET) was used for surface area analysis using Autosorb IQ-XR-XR, Anton Paar, Austria. Thermogravimetry analysis (TGA) was performed using a TGA 4000 from PerkinElmer, Singapore. TA instrument in the temperature range of 50–800 °C under the N_2_ atmosphere at the temperature ramp of 10 °C min^−1^.

### 2.3 Synthetic procedures

#### 2.3.1 Preparation of the catalyst

Two separate processes were employed to prepare kCH catalysts of varying acidity for comparison of their activity, stability, and recyclability.

##### Process A

The commercial κ-carrageenan (white powder, 2.00 g) was taken in a 100 mL beaker. A mixture of 35% aqueous HCl (2 mL) and absolute ethanol (25 mL) was added sequentially to the kC and stirred vigorously at room temperature for 1 h. The suspension was filtered through a Whatman filter paper (grade 5), and the solid residue was washed multiple times with ethanol to remove excess acid. The final product was dried under vacuum for 5 h at RT, yielding kCH-1 as an off-white powder (2.01 g).

##### Process B

Dried κ-carrageenan (2.00 g) was taken in a 100 mL beaker, 27 mL of 0.84 M (concentrated adjusted for comparison with Process A) aq. HCl is added to the kC and kept under vigorous magnetic stirring for 1 h at room temperature. The suspension was filtered through a Whatman filter paper (grade 5), and the solid residue was washed with ethanol to remove excess acid. The final product was dried under vacuum for 5 h at RT, yielding kCH-2 as an off-white powder (1.89 g).

#### 2.3.2 General procedure for the synthesis of furfural acetals

Acetalization of biomass-derived furfurals was performed using suitable diols in the presence of kCH-1 as a heterogeneous acid catalyst. For the synthesis of 2-(furan-2-yl)-1,3-dioxolane (1), furfural (1.001 g, 0.010 mol), ethylene glycol (0.700 g, 0.011 mol), kCH-1 catalyst (0.200 g, 20 wt% of furfural), and cyclohexane (15 mL) were introduced into a round-bottomed flask (100 mL). The flask was fitted with a Dean–Stark apparatus and a water-cooled reflux condenser. The flask was then placed in an oil bath maintained at 110 °C and stirred continuously. During the reaction, water formed as a byproduct distilled off from the reaction vessel, forming an azeotrope with cyclohexane (b.p. 69.8 °C, *χ*(H_2_O) = 0.3). The collected water in the Dean–Stark trap was removed at regular intervals. The reaction was monitored by thin-layer chromatography (TLC) using TLC plates (silica gel coated on aluminium plates) and petroleum ether (60–80 °C)–ethyl acetate (9 : 1, v/v) as the eluent. The KMnO_4_ or 2,4-dinitrophenylhydrazine (DNP) stains were used for visualizing the spots (Fig. S39, SI). After 5 h of reflux, complete conversion of furfural was confirmed by TLC, and the reaction mixture was allowed to cool to room temperature. The reaction mixture was transferred into a centrifuge tube, and the solid kCH-1 catalyst was separated by centrifugation (1000 rpm, 10 min). The liquid was carefully decanted, washed twice with distilled water (10 mL each), and dried over anhydrous sodium sulfate. The solvent was then removed under reduced pressure in a rotary evaporator to afford the acetal product as a light-yellow oil in over 92% yield. The recovered catalyst was washed with ethyl acetate, dried overnight at 60 °C, and reused in subsequent runs.

When the kCH-2 catalyst was used, the reaction kinetics and isolated yield of 1 were comparable. However, the catalyst visibly deteriorated, including partial carbonization, during the reaction.

## 3 Results and discussion

### 3.1 Characterization of the kCH catalysts

The kCH-1 and kCH-2 catalysts were characterised using FTIR, PXRD, BET, and FE-SEM coupled with EDS. The kCH-1 catalyst demonstrated greater structural stability and recyclability than the kCH-2 catalyst, as evidenced by the higher reaction completeness sustained throughout numerous catalytic cycles. Therefore, the catalyst recyclability studies were performed on the kCH-1 system. The fresh and recycled kCH-1 catalysts were characterised to understand their deactivation pathways.

**Fig. 1:**
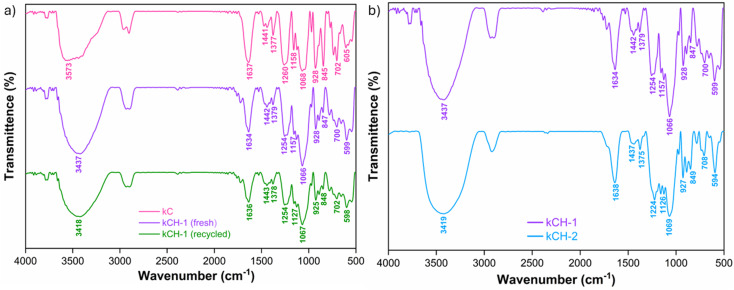
(a) FTIR spectrum of kC, fresh kCH-1, and recycled kCH-1. (b) FTIR spectrum of kCH-1 and kCH-2.

The FTIR spectrum (Fig. 1a) showed all characteristic bands of kC, where a broad peak at 3573 cm^−1^ is due to the OH stretching mode from polysaccharide backbones. The peaks at 1260 cm^−1^ and 845 cm^−1^ represent the characteristic peaks of kC, corresponding to the O=S=O stretching vibration mode and the O–SO_3_ stretching vibration mode at the C4 position of galactose, respectively. The absorbance peak displays the bridge C–O stretching mode at 1158 cm^−1^. The intense peak at 1068 cm^−1^ represents the C–O stretching mode. The characteristic of the C–O–C vibration mode of the 3,6-anhydrogalactose residue absorbance peak appears at 928 cm^−1^. 845 cm^−1^ is the characteristic peak of the galactose 4-sulfate.^40^ The signals at 1441 cm^−1^ and 1377 cm^−1^ are due to the C–O–H in-plane bending vibration mode and C–H bending vibration mode, respectively.^41^ Fresh kCH-1 catalyst showed a peak shift from 3573 cm^−1^ to 3437 cm^−1^, indicating enhanced hydrogen bonding due to protonation. The shift of the asymmetric S=O peak from 1260 cm^−1^ to 1254 cm^−1^ is also possible due to protonation of the sulfate oxygen, resulting in a reduction of electron density. Comparison of the FTIR spectra of kCH-1 and kCH-2 (Fig. 1b) confirms that both samples retain the κ-carrageenan framework, as evident from the fingerprint region. However, noticeable shifts in the O–H and sulfate bands reflect different extents of protonation and hydrogen bonding. The recycled kCH-1 catalyst exhibited a broad peak at 3418 cm^−1^, indicating the retention of hydrogen bonding after recycling. The sulfate ester peak at 1254 cm^−1^ was retained. The presence of 3,6-anhydrogalactose was hinted at by the peak at 925 cm^−1^, suggesting that the core polysaccharide backbone remained intact.

**Fig. 2:**
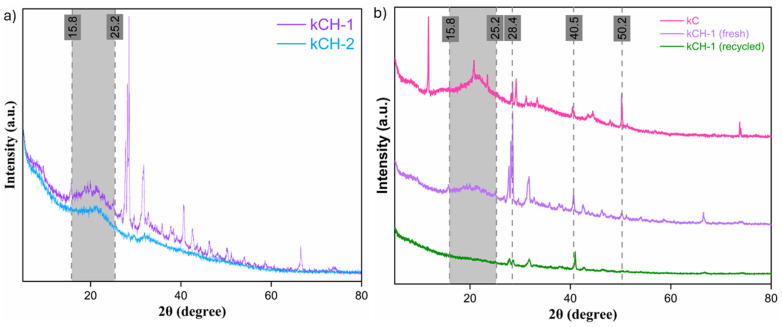
(a) PXRD diffractogram of the two acid-treated catalysts kCH-1 and kCH-2. (b) PXRD diffraction pattern of kC, fresh kCH-1, and recycled kCH-1.

Powder X-ray diffraction (PXRD) analysis of kC, fresh kCH-1, and recycled kCH-1 showed a broad hump in the range of 15.8–25.2° (Fig. 2b), which signifies the semicrystalline characteristic of the sulfated polysaccharide. Sharp peaks at approximately 28.4°, 40.5°, and 50.2° are likely due to inorganic components, such as potassium salts, sodium salts, or other residual minerals.^42^ The sharp peak at 10° in kC arises from regular lateral packing of double helices, which is stabilized by the counter ions, such as K^+^ and Na^+^. The peak disappeared in fresh and recycled kCH-1 due to disruption of the helix aggregation.^43,44^ The intense peak at 28.4° and peaks formed around 28.4° are likely due to interaction between kC and aq. HCl during the preparation of the catalyst. The process adopted for proton exchange of kC by HCl treatment significantly impacts the extent of proton exchange, resulting in a notable difference in the morphology of the catalytic materials. There is a significant difference in the amount of inorganic salts between the two samples. The kCH-1 sample had larger and sharper signature peaks for KCl. In contrast, the recycled kCH-1 catalyst exhibited less intense peaks compared to the fresh kCH-1 catalyst, possibly due to the loss of crystallinity or the formation of amorphous deposits and chemical degradation during the recycling process.

**Fig. 3:**
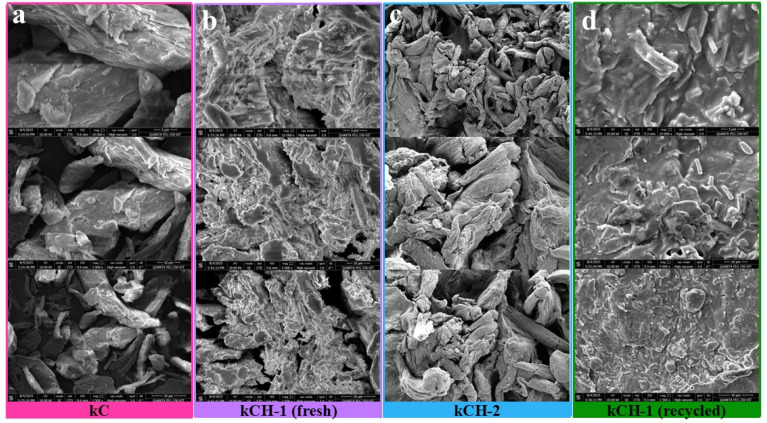
FE-SEM images of: (a) kC, (b) fresh kCH-1, (c) fresh kCH-2, and (d) recycled kCH-1 at the magnification of 5 µm (top), 10 µm (middle), and 30 µm (bottom).

The FE-SEM images depicted in Fig. 3 clarify the distinct morphological transformations upon protonation under different acid-treatment conditions. The FE-SEM images of kC showed dense, large, and relatively smooth structures with compact surface morphology.^45,46^ The fresh kCH-1 catalyst, prepared using a mixture of 35% HCl and ethanol, exhibits a more fragmented and loosely packed structure, with increased surface roughness, cracks, and pores compared to kC. These changes were likely due to the cation exchange during protonation, which disrupted the dense polymer matrix of kC. The recycled kCH-1 catalyst exhibited a more compact surface with a smoother region and showed irregular crystalline deposits, indicating partial structural degradation, which led to morphological changes and reduced porosity. The kCH-2 catalyst prepared by using a purely aqueous 0.84 M HCl solution shows a more fibrous, highly entangled, and densely aggregated morphology, which suggests a stronger acid-induced restructuring of the carrageenan matrix.

**Fig. 4:**
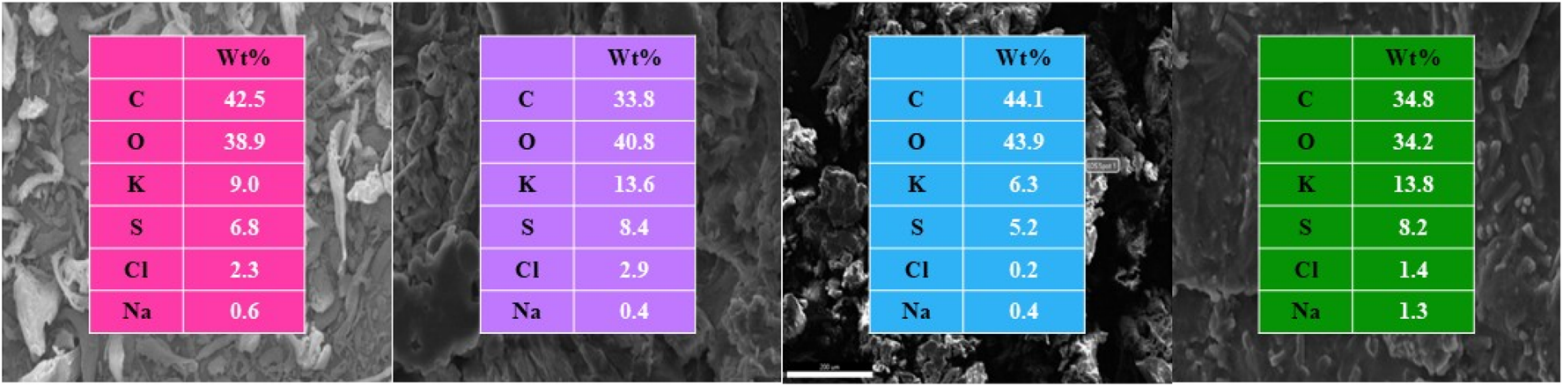
EDS analysis of kC (pink), fresh kCH-1 (purple), kCH-2 (blue), and recycled kCH-1 (green).

The EDS analysis (Fig. 4) of kC, fresh kCH-1, and recycled kCH-1 revealed dominant carbon and oxygen peaks, which are expected from an organic polysaccharide. The sulfur content pointed to the presence of sulfate groups. The elemental composition changed noticeably after HCl treatment and after recycling. In kC, the corresponding peaks for sodium and potassium ions are noticeable, since these counterions balance the sulfate groups in kC. The potassium content was expected to be reduced in the kCH-1 catalyst compared to kC due to protonation. However, a higher amount of K was observed, likely due to the co-precipitation of KCl on the surface of kCH-1 during its preparation. Crystalline KCl gives a strong EDS signal and a sharp XRD diffraction at 28.3° (Fig. 2). In contrast, kCH-2, prepared without ethanol, displayed significantly lower Cl and K contents than kCH-1, suggesting the presence of a lesser amount of KCl. The EDS confirms that the extent of protonation and mineral deposition depends significantly on the catalyst preparation.

Volumetric titration with 0.01 N KOH was used to assess the acid site density of kC, which was observed to steadily rise following HCl treatment. The acidity of the kC was 0.05 mmol g^−1^, while the acidity of the kCH-1 catalyst was 0.13 mmol g^−1^. The acid site density in kCH-2 was significantly higher with a value of 1.12 mmol g^−1^, indicating a nearly tenfold increase in acid sites. The creation of strongly acidic sulfonic acid through the proton exchange of native K^+^/Na^+^ ions introduces acidity into the kCH catalysts. Whereas recycled kCH-1 showed 0.091 mmol g^−1^.

The ammonia temperature-programmed desorption (NH_3_-TPD) analysis of kC, kCH-1, and kCH-2 was performed to check the acid value. However, these materials started to decompose inside the cell during the analysis, resulting in irregular peaks on the graph. However, the acid sites present in kCH-1 and kCH-2 can be observed, whereas they were absent in kC (Fig. S29, SI).

**Fig. 5:**
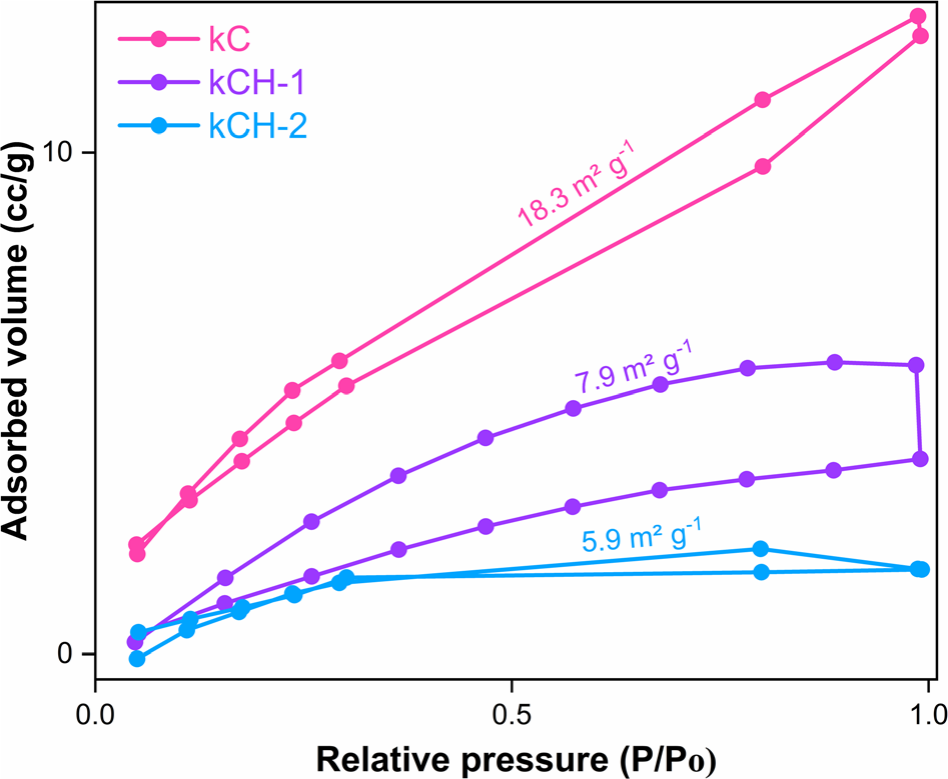
BET analysis of kC, kCH-1, and the kCH-2 catalyst.

The textural properties of the catalysts were investigated by nitrogen adsorption measurements at 77 K. The specific surface area was calculated using the BET method. The BET surface area of kC (pink) was found to be 18.3 m^2^ g^−1^, which decreased to 7.9 m^2^ g^−1^ for kCH-1 (purple) and further to 5.9 m^2^ g^−1^ for kCH-2 (blue) after acid treatment (Fig. 5). This gradual reduction in surface area upon HCl protonation can be attributed to polymer chain rearrangement, partial pore collapse, and aggregation induced by strong hydrogen bonding. The total pore volume was determined at a relative pressure (*p*/*p*_0_) of approximately 0.99. The average pore diameter was obtained from nitrogen adsorption data based on the average pore radius provided by the ASiQwin software (Table 1).

**Table 1:** The BET surface area, total pore volume, and average pore diameter of the kC, kCH-1, and kCH-2 catalyst

Sample	BET area (m^2^ g^−1^)	Total pore volume (cm^3^ g^−1^)	Average pore diameter (nm)
kC	18.3	0.006	3.0
kCH-1	7.9	0.002	4.1
kCH-2	5.9	0.003	1.7

**Fig. 6:**
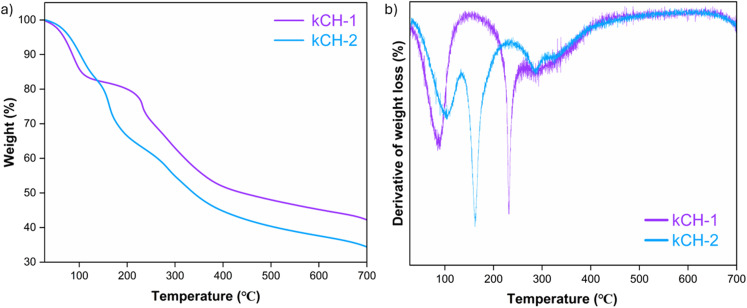
(a) The thermogram of kCH-1 and kCH-2, and (b) DTG plot of kCH-1 and kCH-2.

In both the kCH-1 and kCH-2 catalysts, the TGA data indicated the presence of two major mass-loss events (Fig. 6). The initial event is attributed to the removal of adsorbed and bound water or volatile species, which occurred at temperatures of approximately 87.9 °C (kCH-1, 10.7% loss) and 103 °C (kCH-2, 10.3% loss). The temperature is higher for kCH-2 compared to kCH-1, indicating that water is more strongly bound by the former. The second, much larger event is related to the thermolysis of the polysaccharide backbone (and possible loss of sulphate groups and low-molecular fragments) in the case of kCH-1 (24.93% loss) and in the case of kCH-2 (24.42% loss) at 232 °C and 161 °C, respectively. The higher decomposition temperature of kCH-1 as compared to that of kCH-2 is due to the difference in the extent of proton exchange. A greater extent of proton exchange in kCH-2 autocatalyzes its heat and acid-promoted depolymerization and dehydration reactions. Therefore, kCH-1 exhibited satisfactory thermal stability, which assures its reusability in the acetalization reaction under refluxing cyclohexane.

**Fig. 7:**
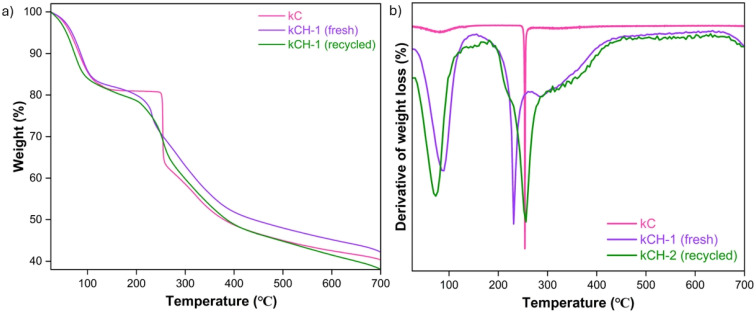
(a) TGA analysis of kC, fresh kCH-1, and recycled kCH-1 catalyst. (b) DTG of kC, fresh kCH-1, and recycled kCH-1 catalyst.

The thermogravimetric analysis (TGA) of kC, fresh kCH-1, and recycled kCH-1 shows different thermal stability (Fig. 7). The native kC displayed two large degradation events: loss of physisorbed and bound water at 80.4 °C (weight loss, 9.23%), and cleavage of sulphate groups and the carrageenan backbone resulted in a second major event at 253.3 °C (weight loss, 20.37%).^41,47^ On protonation, fresh kCH-1 also underwent a two-stage degradation, with the first peak at 87.9 °C (10.7% weight loss) and a second, stronger one at 232.1 °C (24.93% weight loss), indicating that the protonated matrix was more sensitive to thermal conditions. On the other hand, the recycled kCH-1 showed a higher decomposition temperature at 256.5 °C, which resulted in 31.87% weight loss, after an initial dehydration peak at 72.4 °C (9.7% weight loss). The observed behaviour indicates that, even though kCH-1 maintains its general thermal pattern after recycling, the loss of acidity and partial carbonization are responsible for its apparent higher thermal stability.

In a typical process, FF, EG, and kCH-1 were added in cyclohexane, and the suspension was refluxed in a round-bottomed flask. The flask was connected to a Dean–Stark apparatus for azeotropic removal of water. The progress of the reaction was monitored by thin-layer chromatography (TLC) for the disappearance of FF. When the complete conversion of FF was achieved, the reaction mixture was cooled down to RT, filtered to remove the catalyst, washed with saturated NaHCO_3_, and the cyclohexane layer was separated. 2-(Furan-2-yl)-1,3-dioxolane (1) was isolated by evaporating cyclohexane under reduced pressure. Cyclohexane was the most effective of the various water removal agents.

**Fig. 8:**
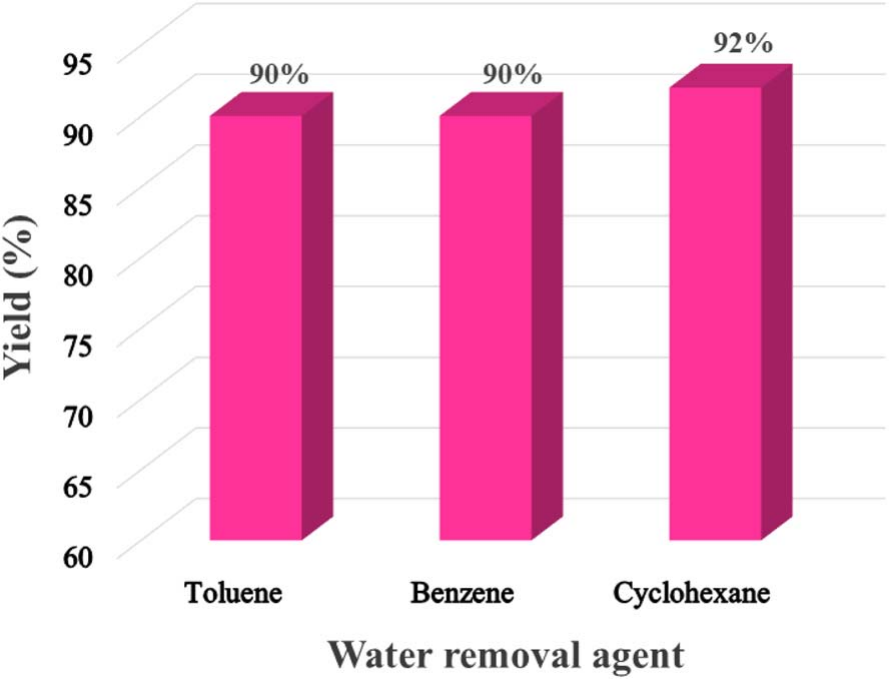
Effect of water removal agent on the preparation of 1.

The acetalization reaction in toluene requires a higher temperature due to the higher boiling point of toluene and the toluene–water azeotrope. Higher temperature degrades the kCH-1 catalyst, making it challenging to recycle. Since cyclohexane is a greener solvent than benzene and toluene and afforded a slightly better yield of acetal 1, it was considered the most suitable solvent and water removal agent for the reaction (Fig. 8).

**Fig. 9:**
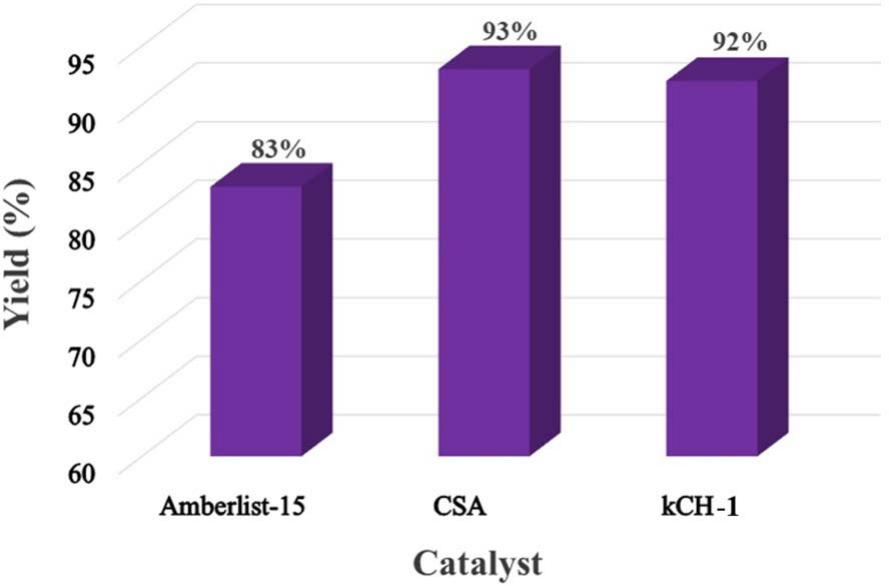
Screening of sulfonic acid-based heterogeneous acid catalysts for preparing 1.

Under optimized conditions, other heterogeneous catalysts with comparable acidity (*i.e.*, sulfonic acid), such as Amberlyst-15 and CSA, were also used, and their catalytic activity was compared with that of kCH-1 (Fig. 9). CSA and kCH-1 catalysts showed good catalytic activity for acetals. However, the conversion of FF was not complete even after extending the reaction duration to 8 h when CSA was used as a catalyst. However, kCH-1 was preferred since its synthesis is more eco-friendly than that of CSA. More specifically, kCH-1 was produced by the simple protonation of kC at ambient conditions, whereas CSA requires the sulfonation of cellulose using concentrated H_2_SO_4_ or chlorosulfonic acid. The control reaction (*i.e.*, without an acid catalyst) yielded only a trace amount of 1 after 8 h at 110 °C.

The molar ratio between FF and EG was varied for faster kinetics and better yields of 1. Typically, an excess of the alcohol reagent is used for the acetalization reaction to favor the equilibrium towards the acetal. However, excess EG complicates the separation of acetal and mandates recycling for better process economy. Therefore, an optimal amount of EG must be used to make the process more reagent-economical without compromising reaction kinetics and product selectivity. When FF and EG were used in an equimolar ratio (1 : 1), a small amount of FF remained unreacted even after 7 h, indicating slower kinetics. In contrast, when the FF : EG molar ratio was increased to 1 : 1.5 with a 20 wt% catalyst loading, the reaction was completed within 5 h, indicating that a slight excess of EG significantly reduces the reaction time. The catalyst loading of kCH-1 was also varied to see the effect on reaction kinetics and product selectivity. At a 10 wt% kCH-1 loading (compared to the mass of FF), the reaction required about 8 h to ensure a quantitative conversion of FF, with the yield of acetal remaining almost unchanged compared to higher loadings. However, when the catalyst loading was increased to 20 wt%, the reaction completed within 5 h, indicating that a higher catalyst concentration accelerates the reaction kinetics without significantly affecting the final yield.

Table 2 shows the molecular structure and isolated yield of the acetals (1–9) starting from FF, MF, and benzaldehyde (BZ) under the optimized reaction conditions. The acetals of FF with EG, 1,2-PDO, and 1,3-PDO afforded excellent isolated yields of the corresponding cyclic acetals (entries 1–3, Table 2). The acetals of MF (entries 4–6, Table 2) were also obtained in satisfactory isolated yields. The substrate scope of the acetalization process was explored by using BZ as the substrate, and no significant different in the selectivity or yield was observed (entries 7–9, Table 2).

**Table 2:** Synthesis of the cyclic acetals of FF, MF, and BZ using protonated κ-carrageenan as the heterogeneous acid catalyst. Reaction conditions: aldehyde (10 mmol), Diol (11 mmol), cyclohexane (15 mL), kCH-1 catalyst (20 wt% of aldehyde), 110 °C, 5 h

Entry	Aldehyde	Polyol	Cyclic acetal	Yield (%)
1	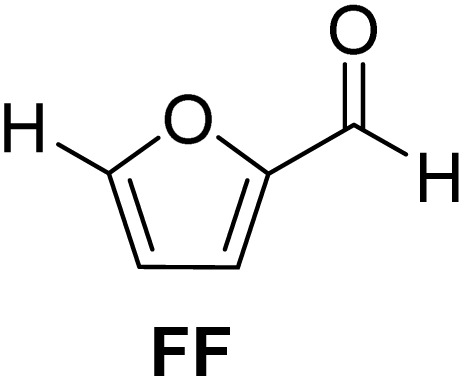	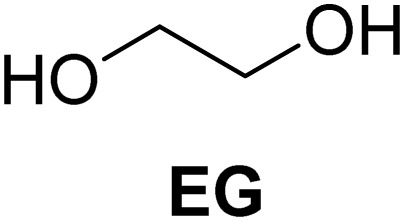	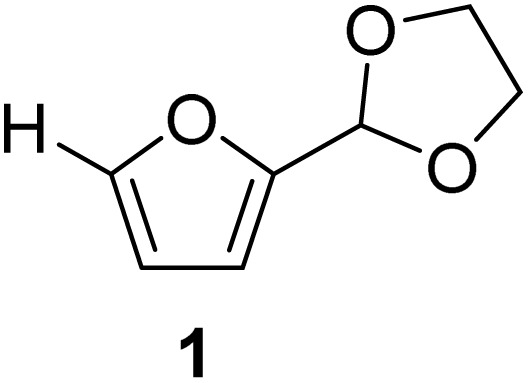	92%
2		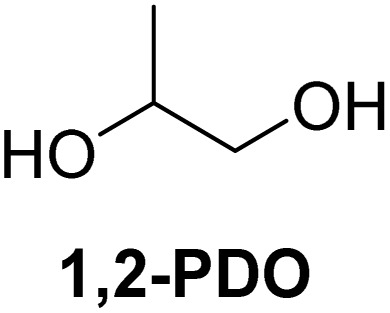	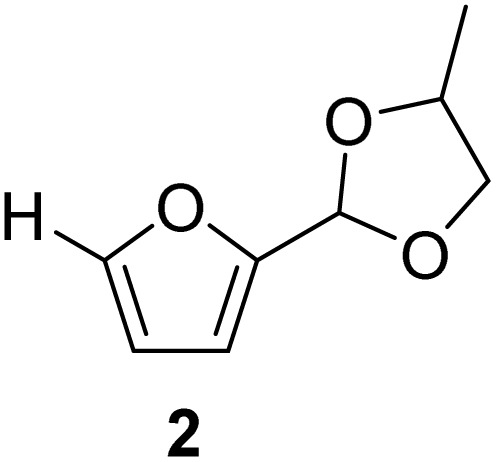	90%
3		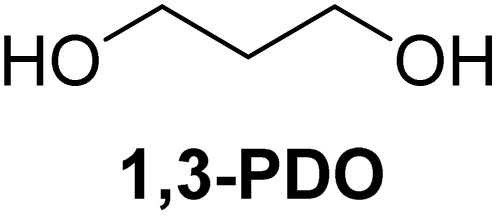	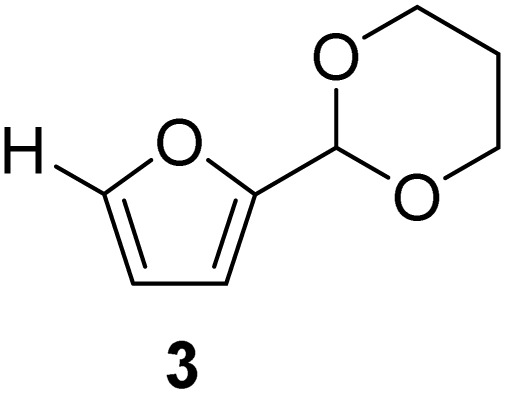	89%
4	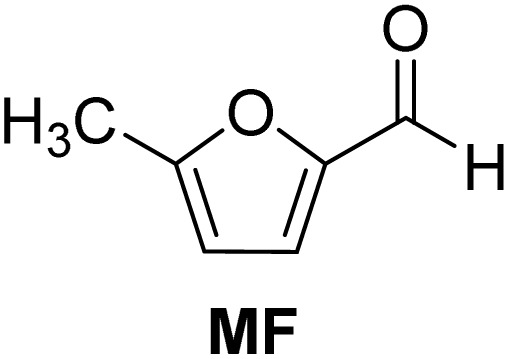	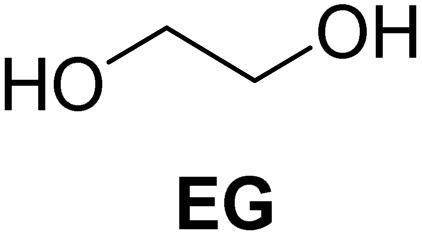	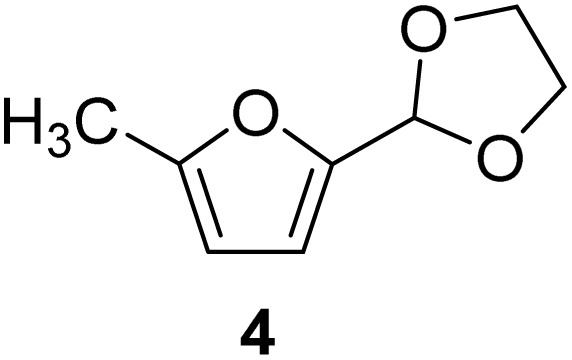	91%
6		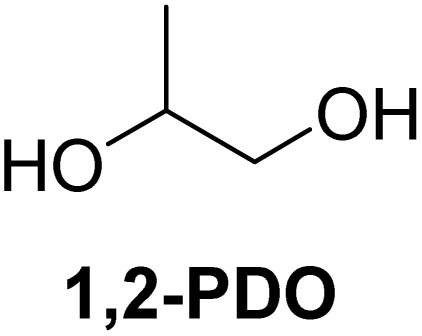	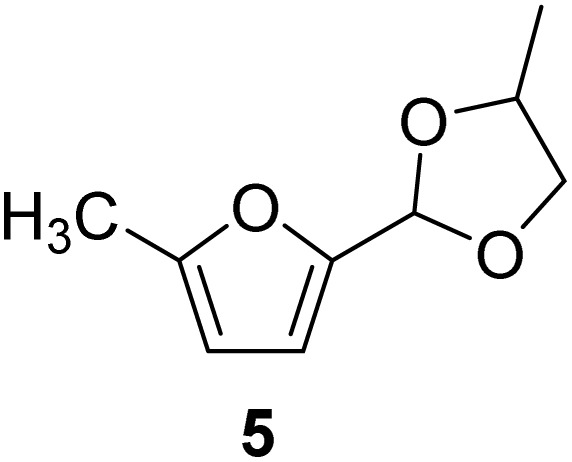	88%
7		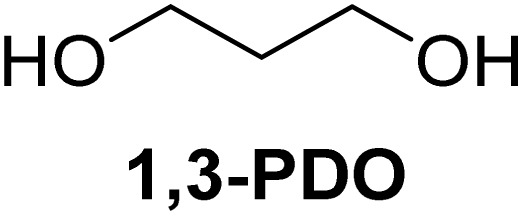	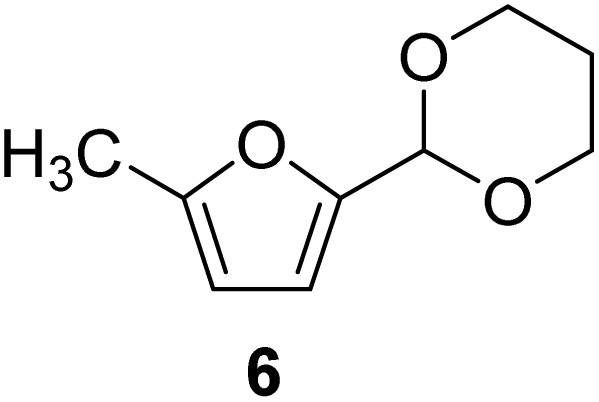	88%
9	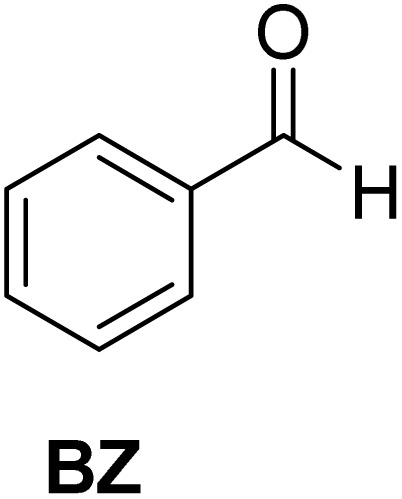	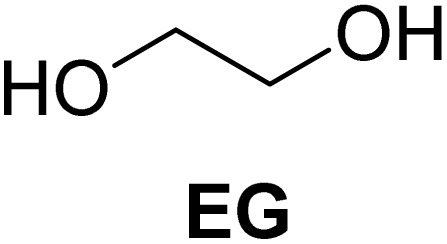	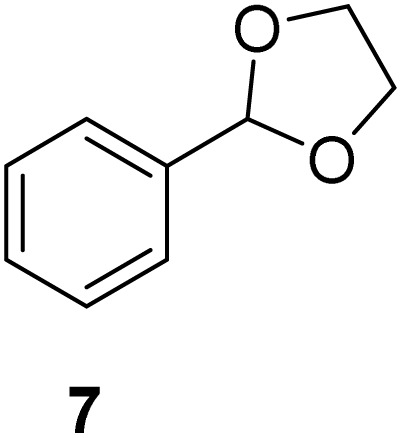	91%
10		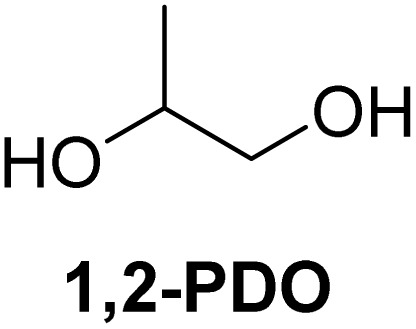	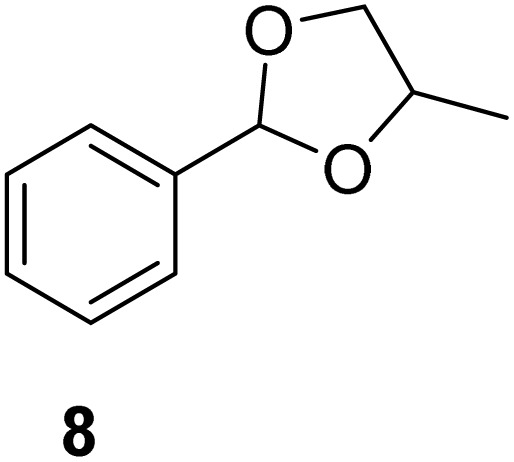	91%
11		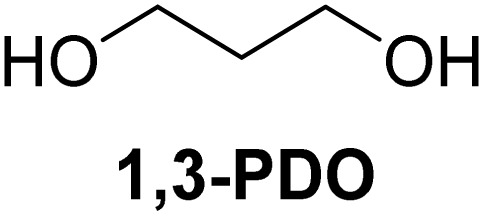	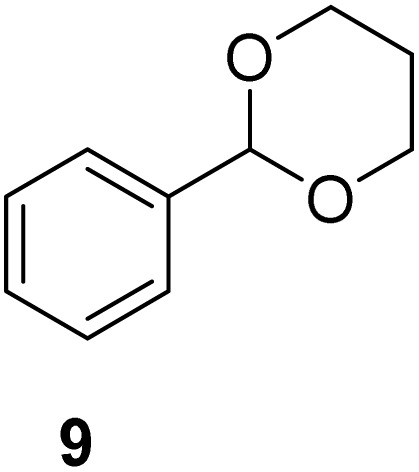	90%

The protonated form of ι-carrageenan was also screened for its catalytic activities. We envisioned that the acid density of ι-carrageenan would be significantly higher than that of kC since the former has two sulfate groups in the repeating unit compared to only one in the latter. The results showed comparable kinetics, yield, and recyclability of protonated ι-carrageenan to those of kCH-1. When purified HMF was used as a substrate for the acetalization reaction with EG, significant decomposition of HMF into insoluble humin was noted, resulting in significant material loss, and only a trace amount of the equivalent acetal was obtained. The observation is commensurate with the poor thermal and acid stability of HMF, which is well-documented in the literature.

## 4 Recyclability of the kCH-1 catalyst

**Fig. 10:**
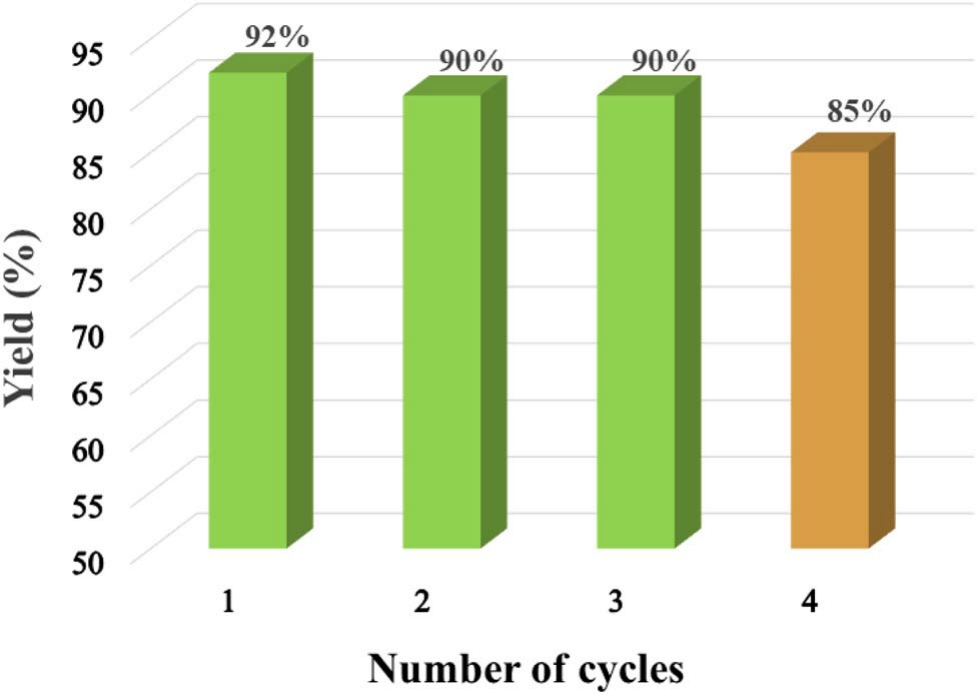
Recyclability of the kCH-1 catalyst for synthesizing 1 (110 °C, 5 h).

Recyclability of the kCH-1 (Fig. 10) catalyst was investigated over four consecutive cycles in the preparation of 2-(furan-2-yl)-1,3-dioxolane (1). The reaction kinetics remained relatively unaltered until the third cycle, indicating that the catalytic efficiency was maintained. However, the conversion of FF was not complete in the fourth cycle, even after an extended duration, hinting at a reduced catalyst activity. However, the yield of 1 at the fourth cycle was still satisfactory (85%). With each recycling, the kCH-1 catalyst became increasingly colored and sticky (semi-solid), indicating partial depolymerization and decomposition.

The recycling of the kCH-2 catalyst was investigated, revealing noticeably lower stability compared to the kCH-1 catalyst. Partial decomposition of the kCH-2 catalyst resulted in incomplete conversion of FF in the second cycle. Therefore, further recycling attempts of the kCH-2 catalyst were not undertaken. An attempt was made to initially prepare the kCH-2 catalyst by treating kC with 35% aqueous HCl (2 g kC with 6 mL HCl) and washing the filtered solid with cold water to remove excess HCl. However, the catalyst began to carbonize even at room temperature during the storage and drying process. Therefore, kCH-2 was prepared using dil. HCl and the recyclability of the kCH-2 catalyst were investigated. However, the kCH-2 catalyst prepared in this way showed noticeably lower stability compared to the kCH-1 catalyst. Partial decomposition of the kCH-2 catalyst resulted in incomplete conversion of FF in the second cycle. Therefore, further recycling attempts of the kCH-2 catalyst were not undertaken.

The impact of the extent of protonation of kC on its stability and recyclability was further confirmed. Specifically, the kCH-2 catalyst was treated with an aqueous solution of KCl and then dried. The storage stability improved, and no color change was observed even after 2 days of storage at RT. The recyclability of the catalyst was evaluated over four cycles, yielding a similar pattern to that of kCH-1 (Fig. S33, SI). The observation can be explained by the lowering of the acidity of kCH-2 by K^+^ in KCl, which replaces some protons, thereby increasing storage and thermal stability while improving recyclability. However, attempts to dry the catalyst at 60 °C resulted in partial decomposition, leading to a darkening of the color (Fig. S34, SI). Therefore, the catalyst was instead dried under vacuum at RT.

## 5 Conclusion

In conclusion, protonated κ-carrageenan was used as a marine biomass-derived renewable biopolymer-derived heterogeneous acid catalyst for the acetalization of carbohydrate-derived furfural and 5-methylfurfural. Ethylene glycol, 1,2-propanediol, and 1,3-propanediol were used as the diol reagents. Since the diols mentioned above can be renewably sourced from carbohydrates, this work expands the scope of employing biogenic carbon atoms in synthesizing value-added organic chemicals in a carbohydrate-centric biorefinery, where the substrate, reagent, and catalyst are sourced from carbohydrates. The reactions were performed in a Dean–Stark apparatus using cyclohexane as the solvent for water removal. Excellent isolated yields of the corresponding acetals (>90%) were obtained under optimized conditions. The broad substrate scope of the synthetic protocol was successfully demonstrated by preparing the acetals of benzaldehyde. Moreover, the catalyst was successfully recovered and reused for multiple cycles without a catastrophic drop in its catalytic efficiency. The future work will emphasize on the energy efficiency and detailed life-cycle analysis of the feedstock, reagents, and solvents employed in the synthetic process for quantitative evaluation of the environmental sustainability.

## Author contributions

Rachitha S. N. performed the experiments and analyzed the synthetic and spectroscopic data. Saikat Dutta conceptualized the idea, supervised the work, and wrote the original manuscript.

## Conflicts of interest

The authors declare no competing interests.

## Abbreviations

NH_3_-TPD: Ammonia temperature-programmed desorption
BET: Brunauer–Emmett–Teller
kC: κ-Carrageenan
CSA: Cellulose sulfuric acid
EDS: Energy dispersive spectroscopy
EG: Ethylene glycol
FE-SEM: Field-emission scanning electron microscopy
FTIR: Fourier transform infrared
FF: Furfural
GLY: Glycerol
HMF: 5-(Hydroxymethyl)furfural
MF: 5-Methylfurfural
NMR: Nuclear magnetic resonance
PXRD: Powder X-ray diffraction
1,2-PDO: 1,2-Propanediol
1,3-PDO: 1,3-Propanediol
kCH: Protonated κ-carrageenan
TLC: Thin-layer chromatography

## Supplementary Material

RA-016-D5RA10090A-s001

## Data Availability

Supplementary information (SI): The spectroscopic (FTIR, ^1^H-NMR, and ^13^C-NMR) characterization of all synthesized compounds reported in this manuscript. Photographic images of the reaction setup, EDS data, NH_3_-TPD data, TLC images, and images of the fresh and recycled catalysts have also been added. See DOI: https://doi.org/10.1039/d5ra10090a.
