# Supplementary material for: High-yielding and scalable synthesis of furfural acetals using protonated κ-carrageenan as a biorenewable acid catalyst
Source: RSC Adv. 2026 Jan 22;16(6):4776–87. doi: 10.1039/d5ra10090a (PMC12825003; doi:10.1039/d5ra10090a)
Supplement: RA-016-D5RA10090A-s001 [file RA-016-D5RA10090A-s001.pdf]

Supplementary Information (SI) for RSC Advances.
This journal is © The Royal Society of Chemistry 2026

High-yielding and scalable synthesis of furfural acetals using protonated κ-carrageenan as a biorenewable acid catalyst

Rachitha S Natraj,[1] and Saikat Dutta[1*]

[1] Department of Chemistry, National Institute of Technology Karnataka (NITK), Surathkal, Mangalore–575025, Karnataka, India.

[*] Corresponding author. E-mail: sdutta@nitk.edu.in

Number of figures: 39

Number of pages: 22

Spectroscopic (^{1}H NMR, ^{13}C NMR, and FTIR) characterization data of the synthesized acetals

2-(furan-2-yl)-1,3-dioxolane (**1**): Light yellow liquid (92%), ^{1}H NMR ($CDCl_3$, 400 MHz): δ 7.34 (d, 1H,), 6.36 (d, 1H), 6.27-6.26 (m, 1H), 5.83 (s, 1H); ^{13}C NMR ($CDCl_3$, 100 MHz): δ 150.9, 143.0, 110.0, 108.6, 97.6, 65.0; FTIR (ATR, cm^{-1}): 2959, 2892, 1606, 1092.[1]

2-(furan-2-yl)-4-methyl-1,3-dioxolane (**2**): Light yellow liquid (90%), ^{1}H NMR ($CDCl_3$, 400 MHz): δ 7.42-7.40 (d, 1H), 6.46-6.40 (m, 1H), 6.33 (s, 1H), 6.01-5.88 (m, 1H), 4.41-4.05 (m, 2H), 3.60-3.47 (m, 1H), 1.38-1.30 (dd, 3H, J = 6 Hz, J = 6 Hz); ^{13}C NMR ($CDCl_3$, 100 MHz): δ 151.4, 150.9, 142.9, 142.8, 109.9, 109.8, 108.6, 108.1, 97.6, 97.1, 73.3, 72.1, 71.33, 70.7, 17.7, 17.6; FTIR (ATR, cm^{-1}): 2978, 2880, 1603, 1066. [1]

2-(furan-2-yl)-1,3-dioxane (**3**): Light yellow liquid (89%), ^{1}H NMR ($CDCl_3$, 400 MHz): δ 7.31-7.30 (m, 1H), 6.35-6.34 (d, 1H), 6.27-6.26 (m, 1H), 5.48 (s, 1H) 4.15-4.10 (m, 2H), 3.87-3.80 (m, 2H), 2.16-2.06 (m, 1H), 1.34-1.29 (m, 1H); ^{13}C NMR ($CDCl_3$, 100 MHz): δ 150.7, 142.0, 109.7, 106.9, 95.6, 66.8, 25.1; FTIR (ATR, cm^{-1}): 2965, 2854, 1668, 1001. [1]

2-(5-methylfuran-2-yl)-1,3-dioxolane (**4**): Light yellow liquid (91%), ^{1}H NMR ($CDCl_3$, 400 MHz): δ 6.17 (s, 1H), 5.78 (s, 1H), 5.66 (s, 1H), 3.92-3.91 (m, 2H), 3.77-3.74 (m, 1H), 2.12 (s, 3H); ^{13}C NMR ($CDCl_3$, 100 MHz): δ 152.4, 148.8, 109.3, 105.7, 97.3, 64.5, 12.9; FTIR (ATR, cm^{-1}): 2956, 2889, 1674, 1020.

4-methyl-2-(5-methylfuran-2-yl)-1,3-dioxolane (**5**): Light yellow liquid (88%), ^{1}H NMR ($CDCl_3$, 400 MHz): δ 6.21-6.16 (m, 1H), 5.81-5.67 (m, 2H), 4.27-3.88 (m, 2H), 3.45-3.30 (m, 1H), 2.1 (s, 3H), 1.23-1.15 (dd, 3H, J = 4 Hz, J = 6 Hz); ^{13}C NMR ($CDCl_3$, 100 MHz): δ 152.3, 152.2, 149.2, 148.7, 109.3, 108.9, 105.6, 105.5, 97.4, 96.8, 72.7, 71.6, 70.9, 70.3, 17.4, 17.3, 12.9; FTIR (ATR, cm^{-1}): 2978,2876, 1676, 1016.

2-(5-methylfuran-2-yl)-1,3-dioxane (**6**): Light yellow liquid (88%), ^{1}H NMR ($CDCl_3$, 400 MHz): δ 6.27-6.26 (d, 1H), 5.91 (s, 1H), 5.47 (s, 1H), 4.18-4.14 (m, 2H) 3.91-3.85 (m, 2H), 2.25 (s, 3H), 1.37-1.19 (m, 2H); ^{13}C NMR ($CDCl_3$, 100 MHz): δ 151.3, 148.8, 107.5, 105.4, 95.4, 66.4, 24.9, 12.6; FTIR (ATR, cm^{-1}): 2964, 2853, 1675, 1026.

2-phenyl-1,3-dioxolane (**7**): Colorless liquid (91%), ^{1}H NMR ($CDCl_3$, 400 MHz): δ 7.46-7.43 (m, 2H), 7.28-7.25 (m, 3H), 5.71-5.40 (m, 1H), 4.14-3.78 (m, 4H); ^{13}C NMR ($CDCl_3$, 100 MHz): δ 151.3, 148.8, 107.5, 105.4, 95.4, 66.4, 24.9, 12.6; FTIR (ATR, cm^{-1}): 2964, 2853, 1675, 1026.

4-methyl-2-phenyl-1,3-dioxolane (**8**): Light yellow liquid (91%), ^{1}H NMR ($CDCl_3$, 400 MHz): δ 7.46-7.42 (m, 2H), 7.29-7.25 (m, 3H), 5.8-5.7 (m, 1H), 4.17-3.86 (m, 2H), 3.43-3.33 (m, 1H), 1.21-1.14 (dd, 3H, J = 6 Hz, J = 6 Hz); ^{13}C NMR ($CDCl_3$, 100 MHz): δ 138.2, 137.6, 128.4, 128.2, 127.5, 126.0, 125.7, 103.2, 102.2, 72.6, 71.5, 71.1, 70.5, 17.8, 17.6; FTIR (ATR, cm^{-1}): 2976, 2874, 1724, 1025, 1004.

2-phenyl-1,3-dioxane (**9**): Light yellow liquid (90%), ^{1}H NMR ($CDCl_3$, 400 MHz): δ 7.48-7.46 (m, 2H), 7.34-7.29 (m, 3H), 5.45 (s, 1H), 4.22-4.18 (m, 2H) 3.94-3.88 (m, 2H), 2.20-2.11 (m, 1H), 1.36-1.33 (m, 1H); ^{13}C NMR ($CDCl_3$, 100 MHz): δ 138.4, 128.1, 127.6, 125.5, 100.9, 66.7, 25.2; FTIR (ATR, cm^{-1}): 2959, 2854, 1735, 1047.

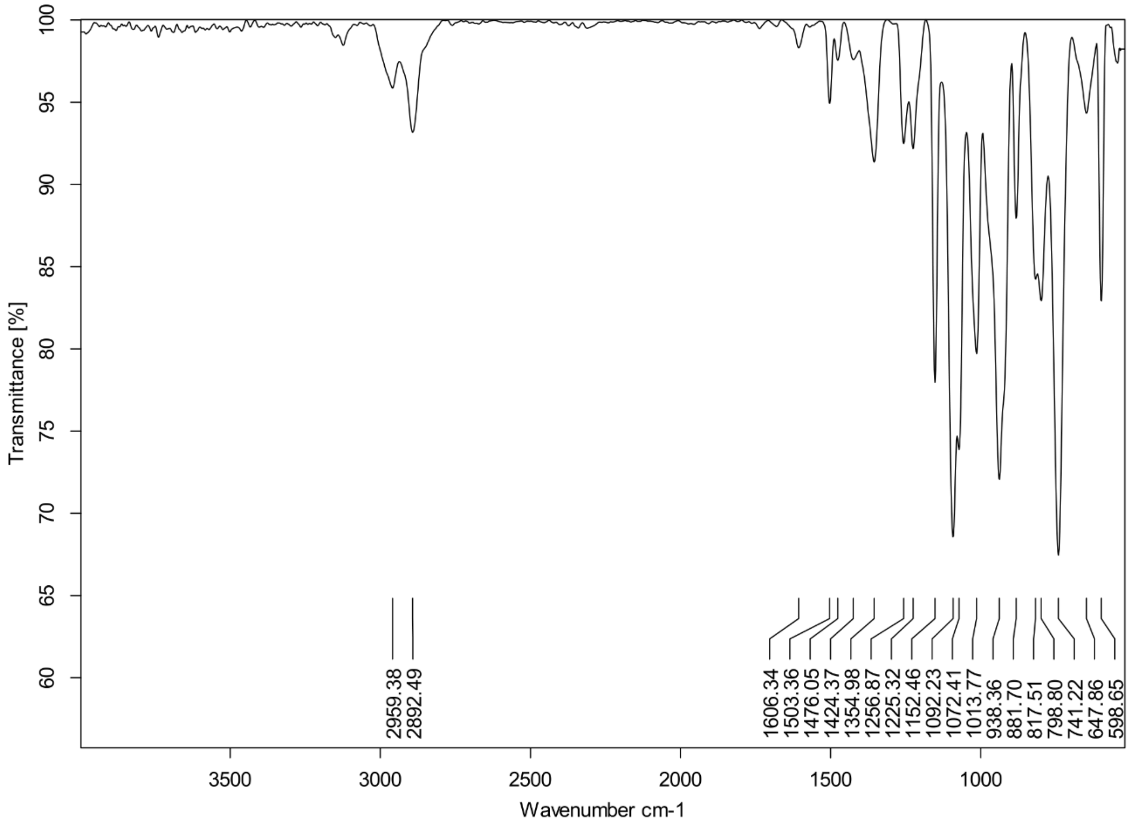

Figure S1. The FTIR spectrum of **1.**

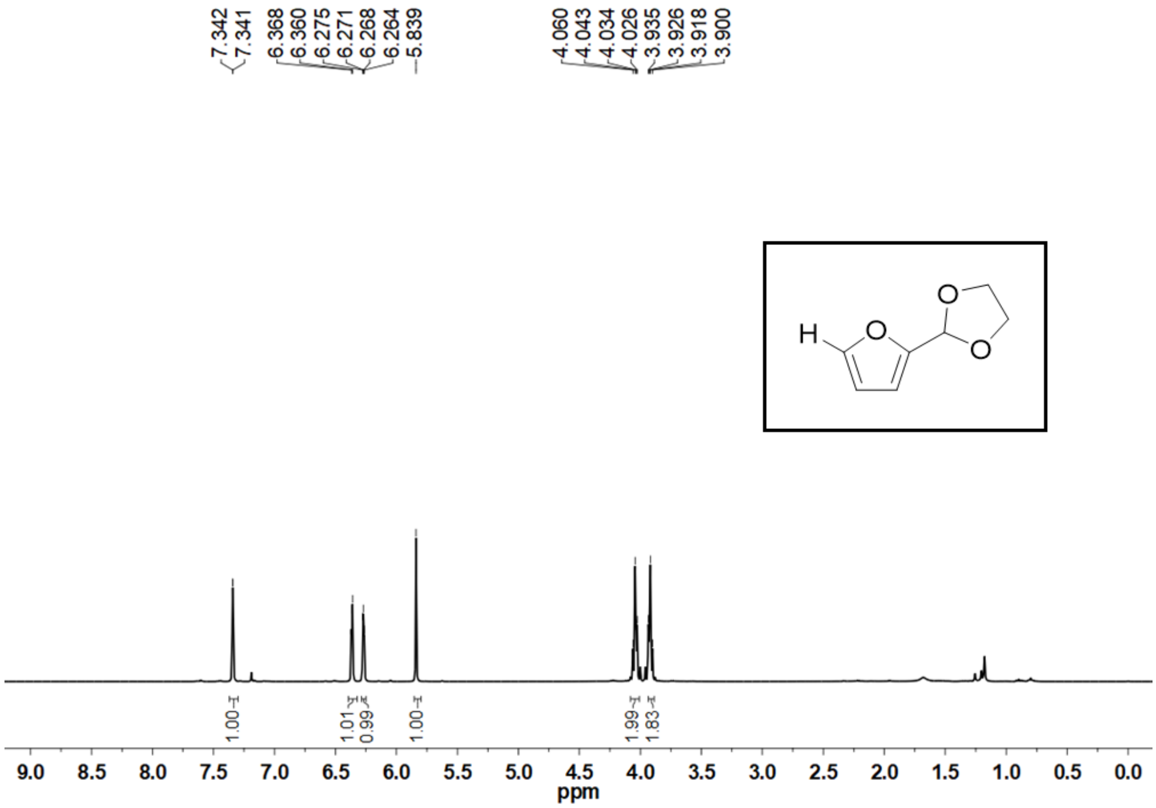

Figure S2. The ^{1}H-NMR spectrum of **1**.

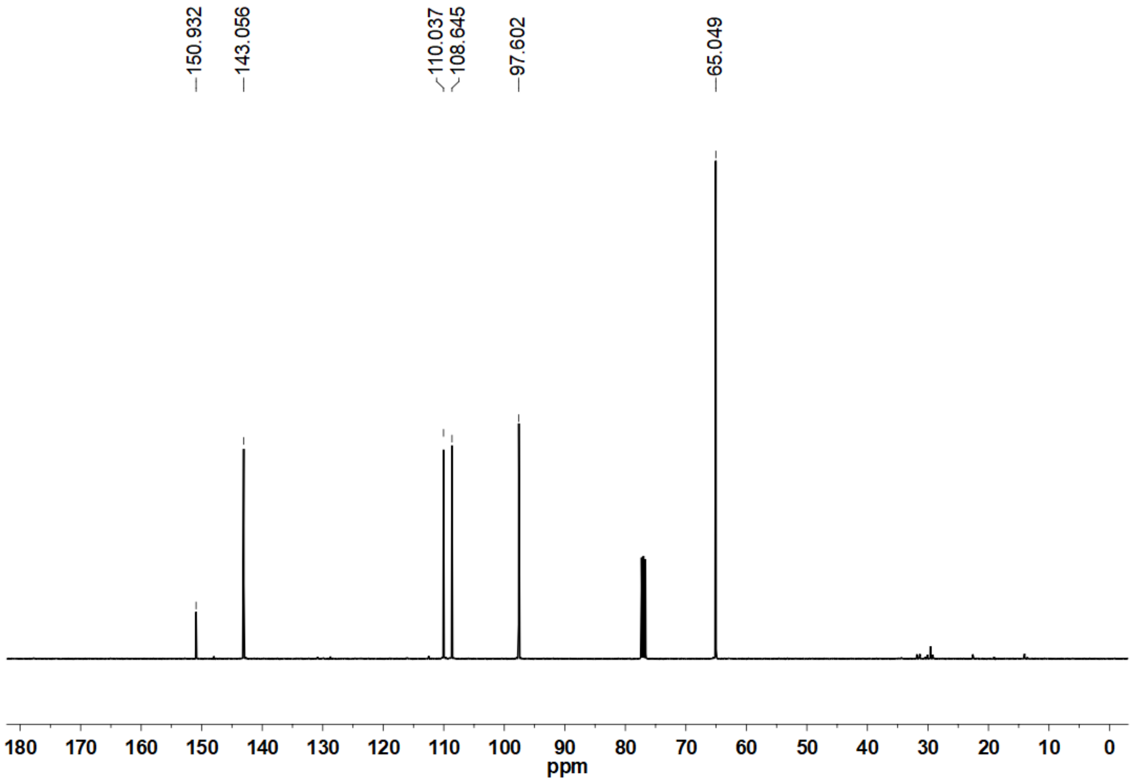

Figure S3. The ^{13}C-NMR spectrum of **1**.

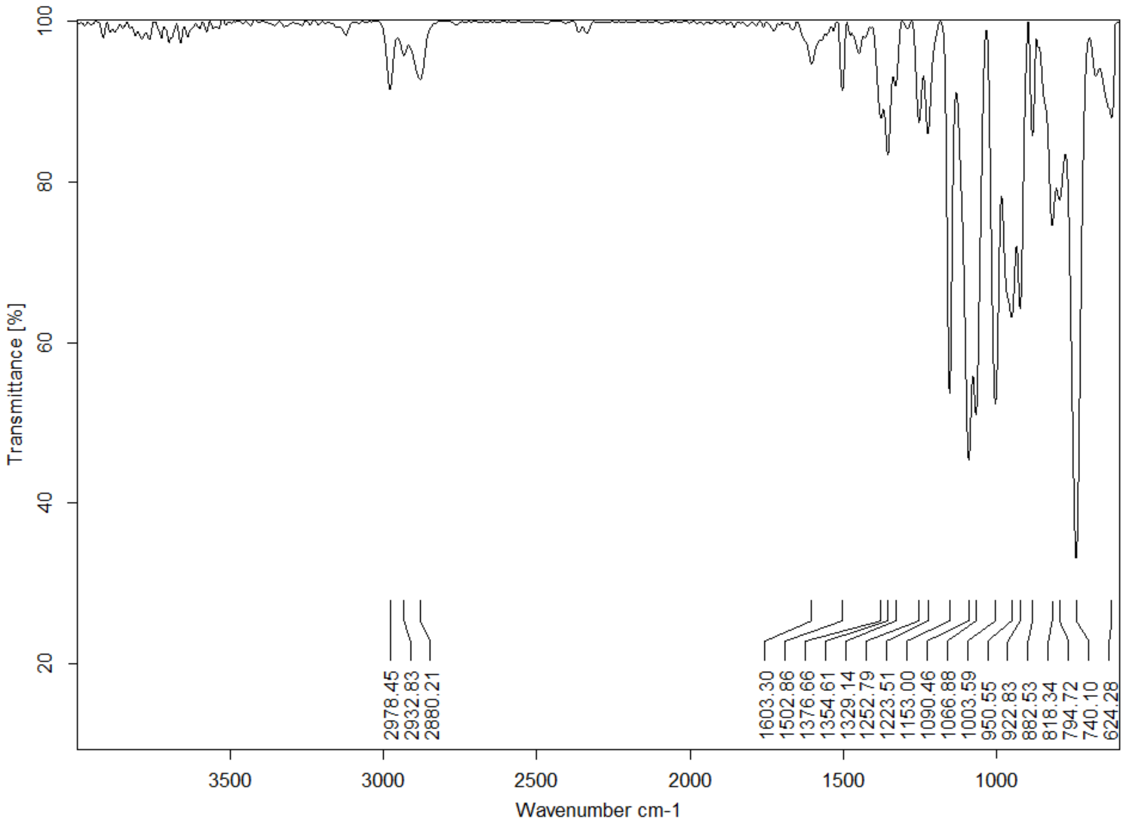

Figure S4. The FTIR spectrum of **2.**

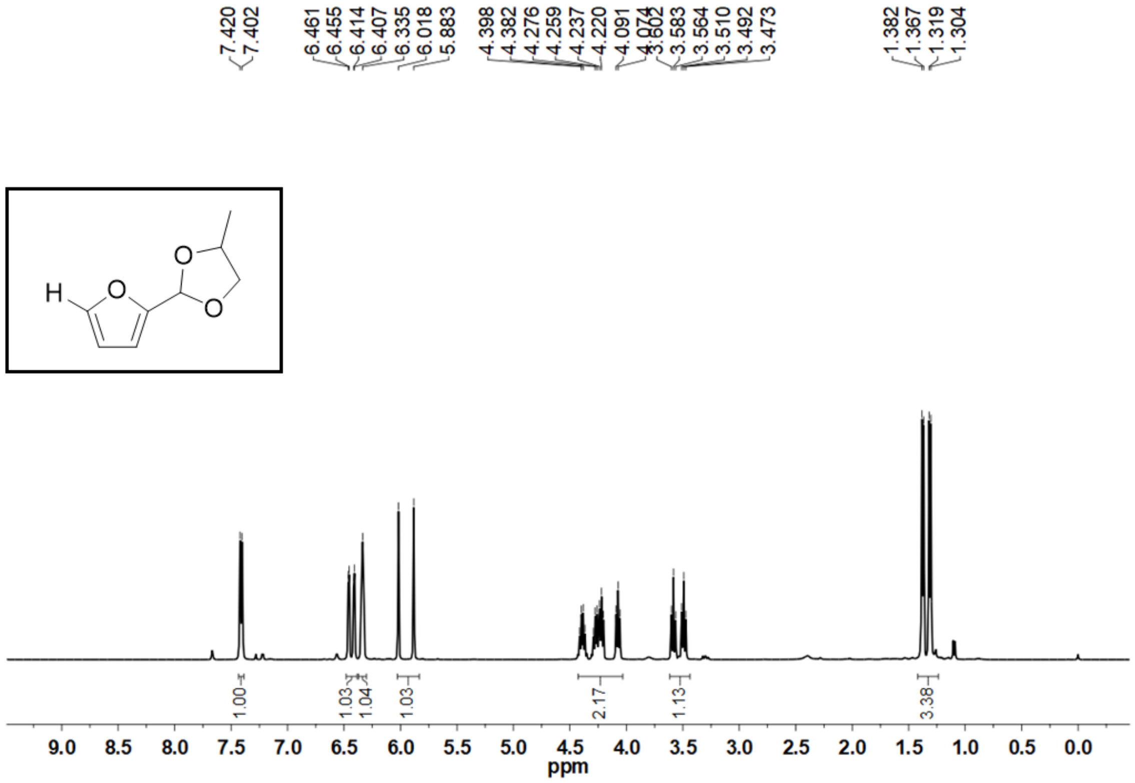

Figure S5. The ^{1}H-NMR spectrum of **2**.

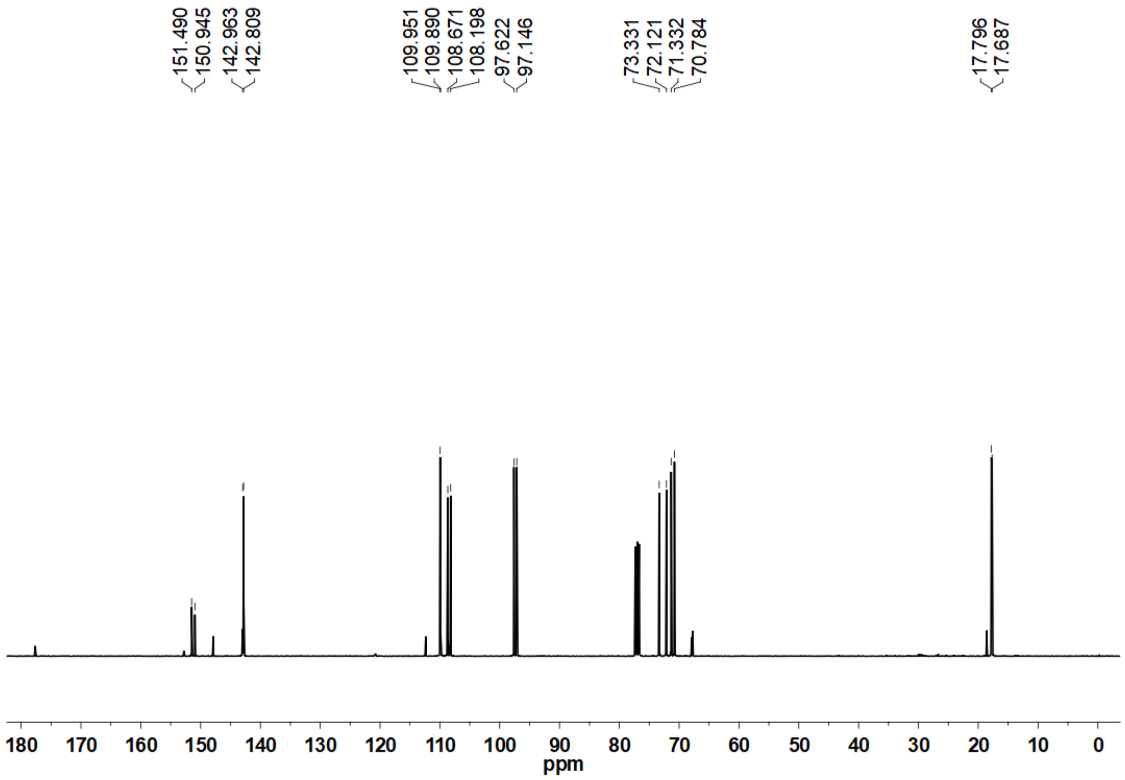

Figure S6. The ^{13}C-NMR spectrum of **2**.

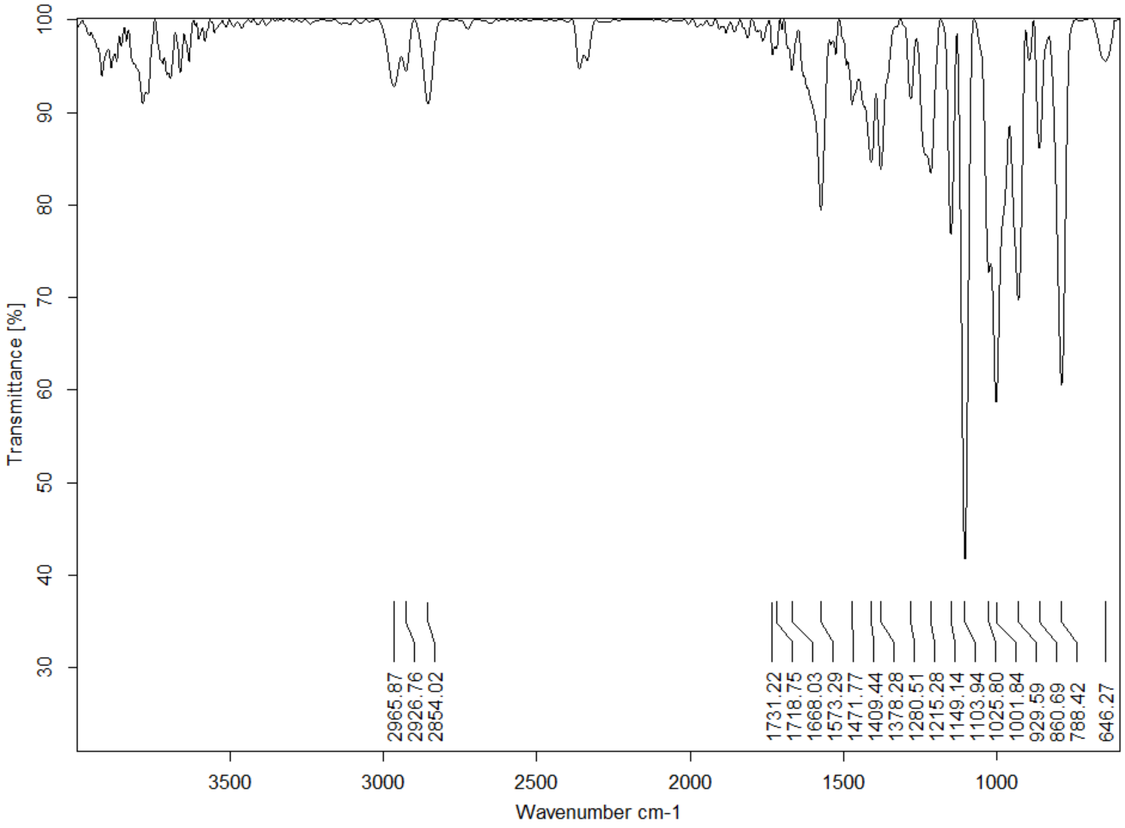

Figure S7. The FTIR spectrum of **3.**

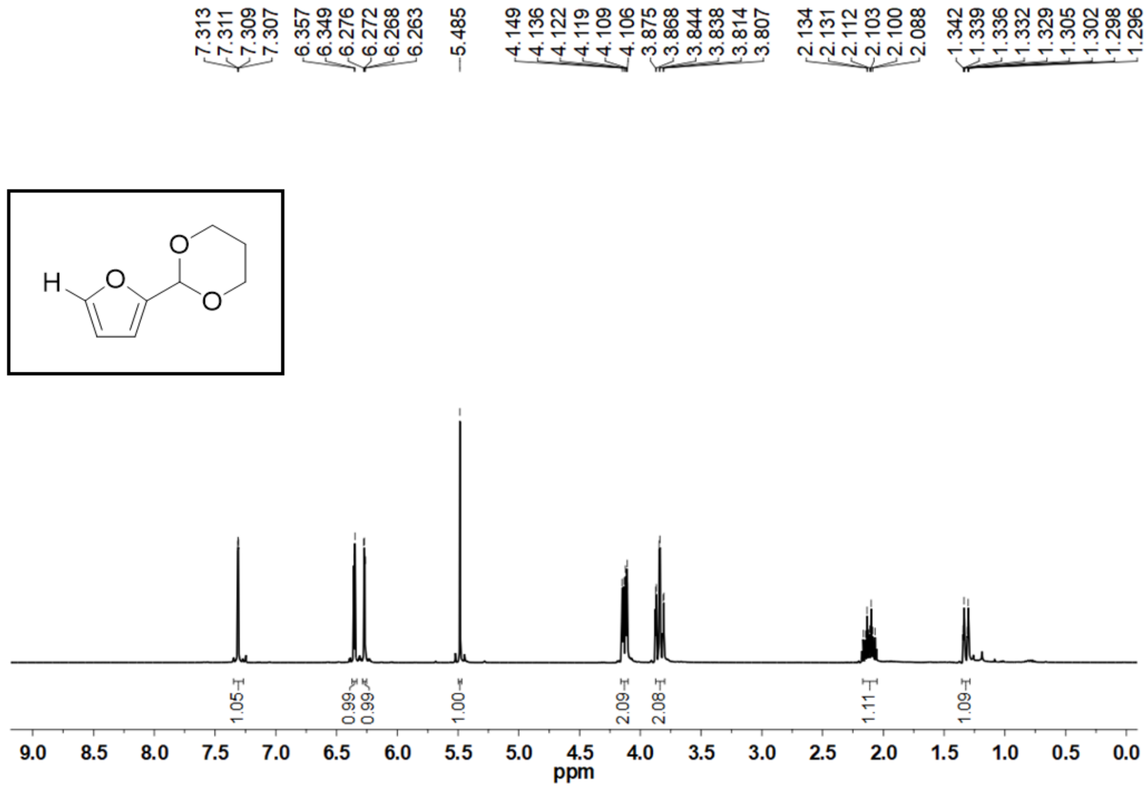

Figure S8. The ^{1}H-NMR spectrum of **3**.

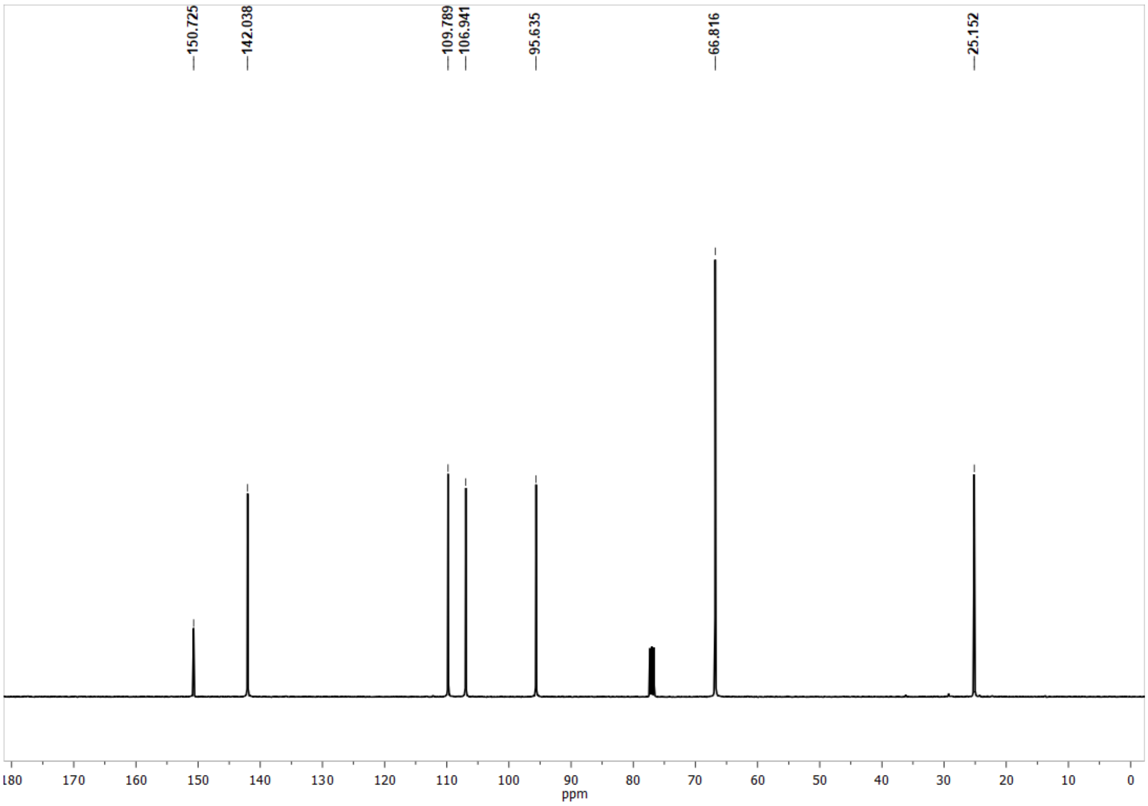

Figure S9. The ^{13}C-NMR spectrum of **3**.

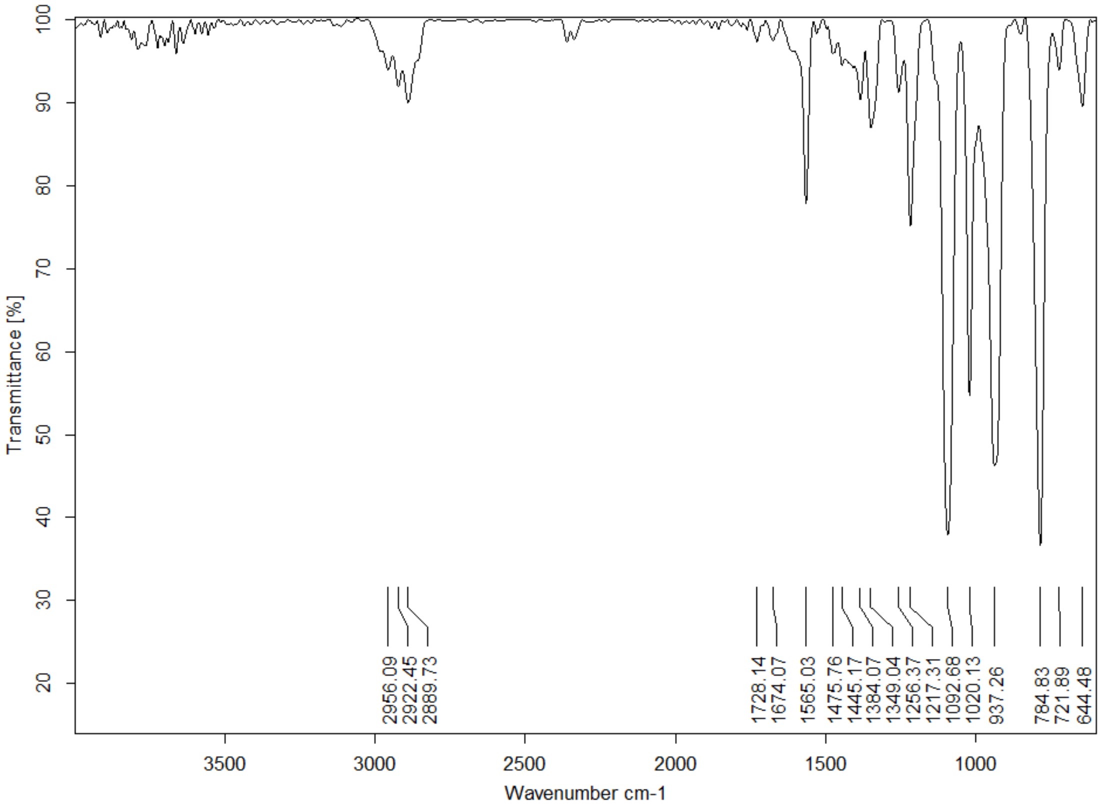

Figure S10. The FTIR spectrum of **4.**

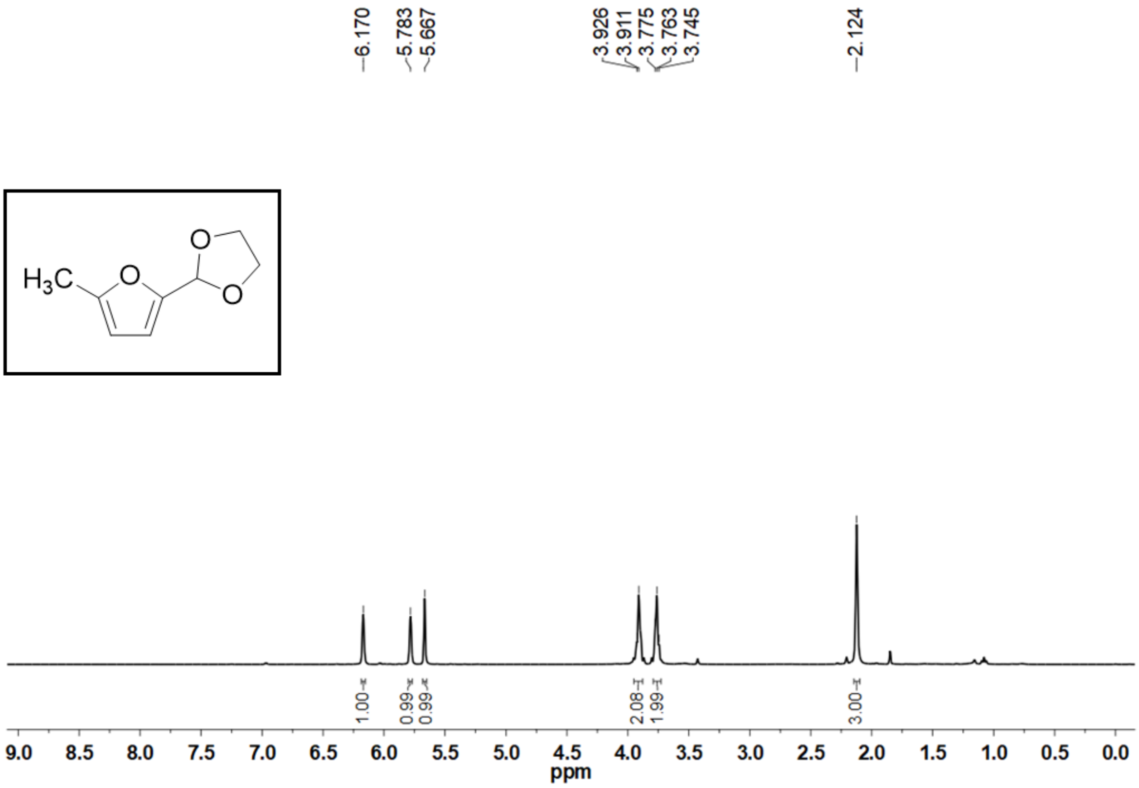

Figure S11. The ^{1}H-NMR spectrum of **4**.

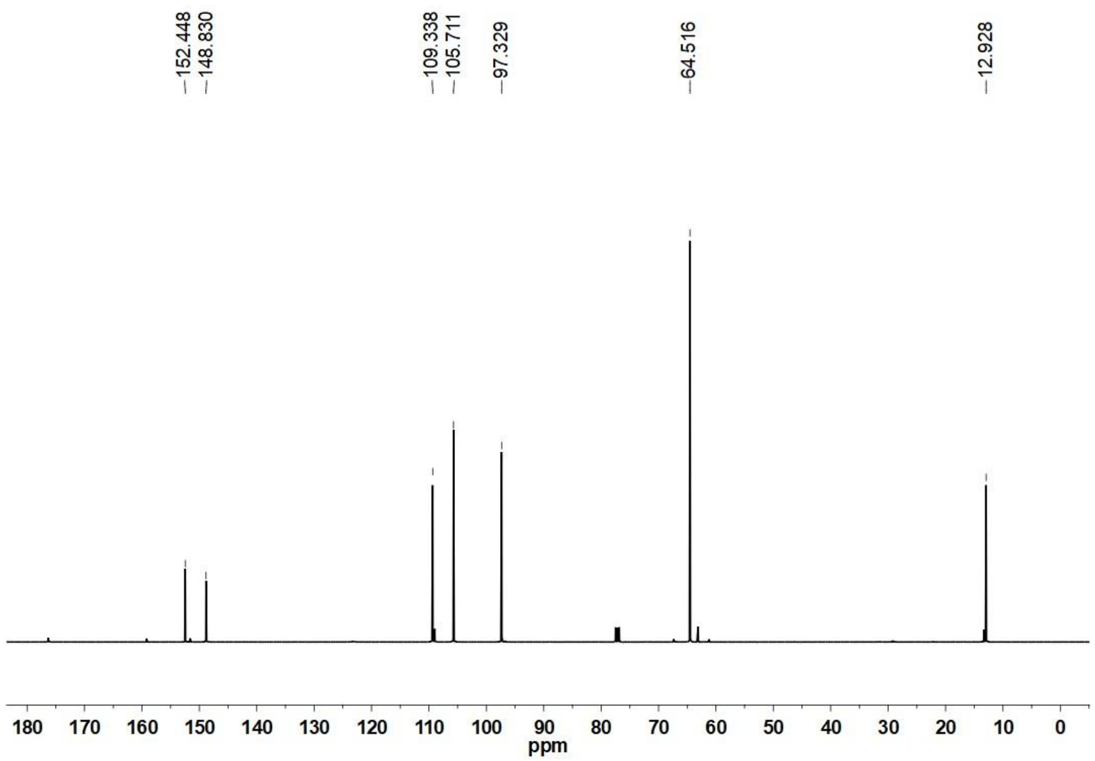

Figure S12. The ^{13}C-NMR spectrum of **4**.

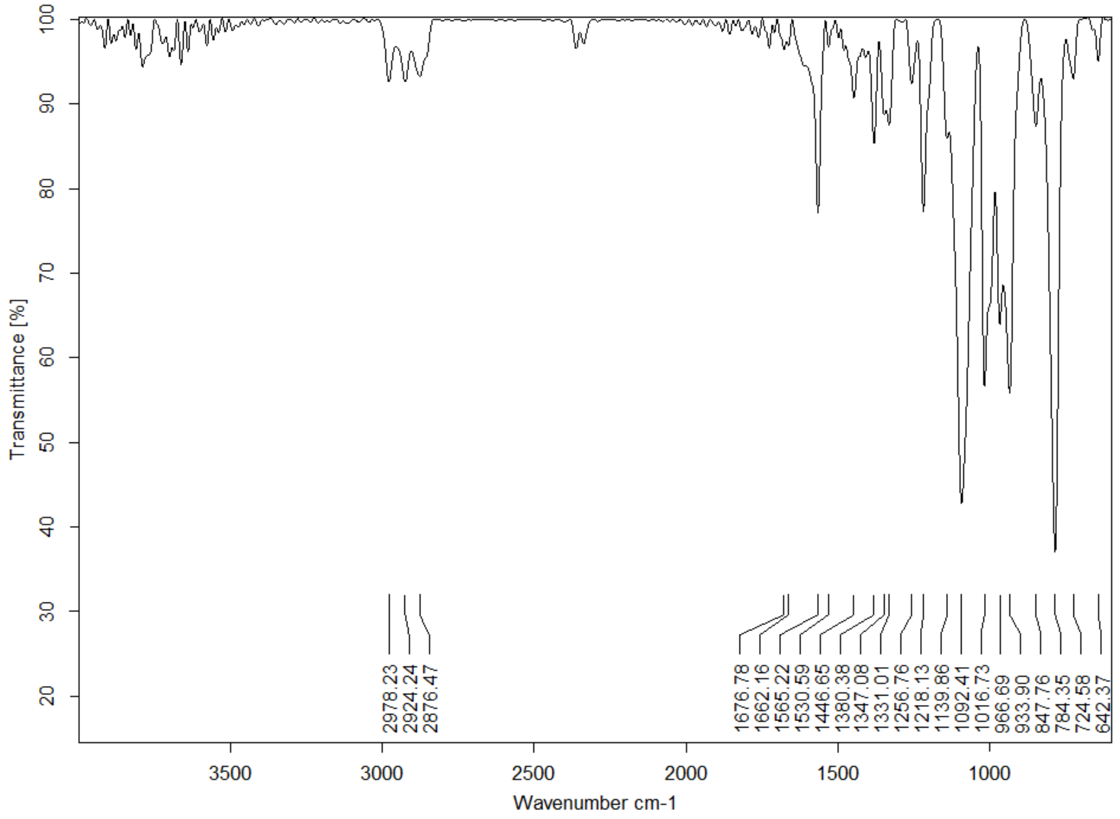

Figure S13. The FTIR spectrum of **5.**

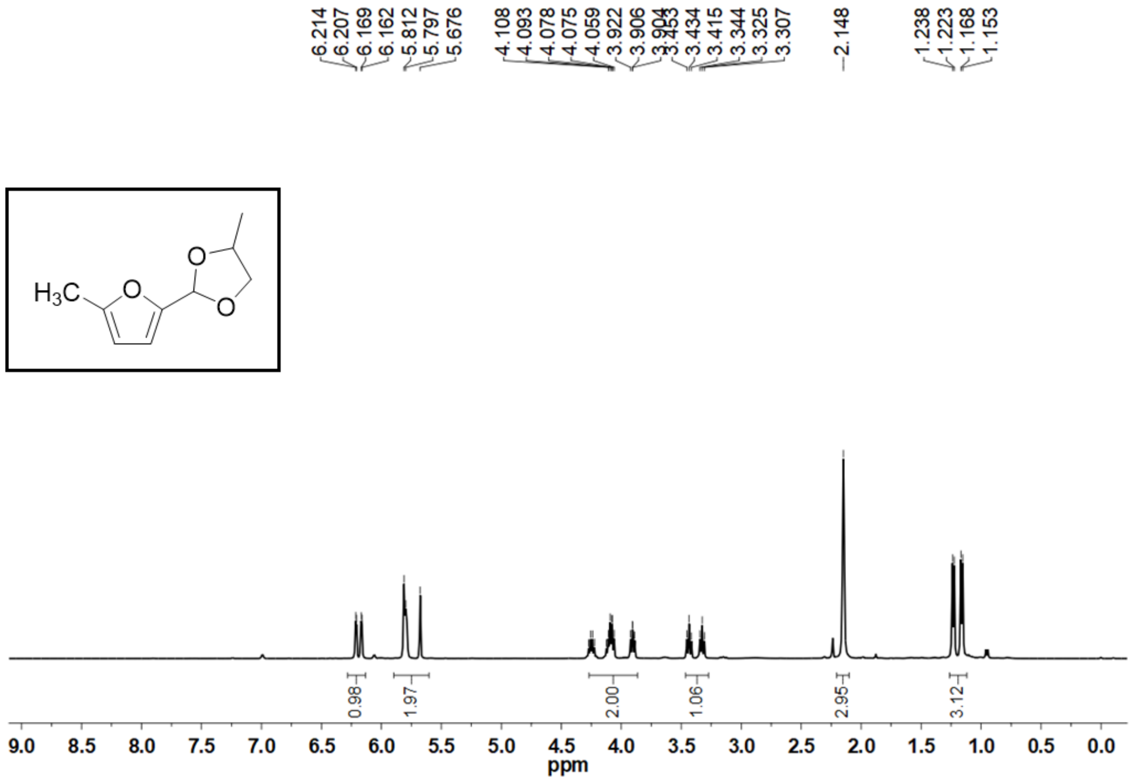

Figure S14. The ^{1}H-NMR spectrum of **5**.

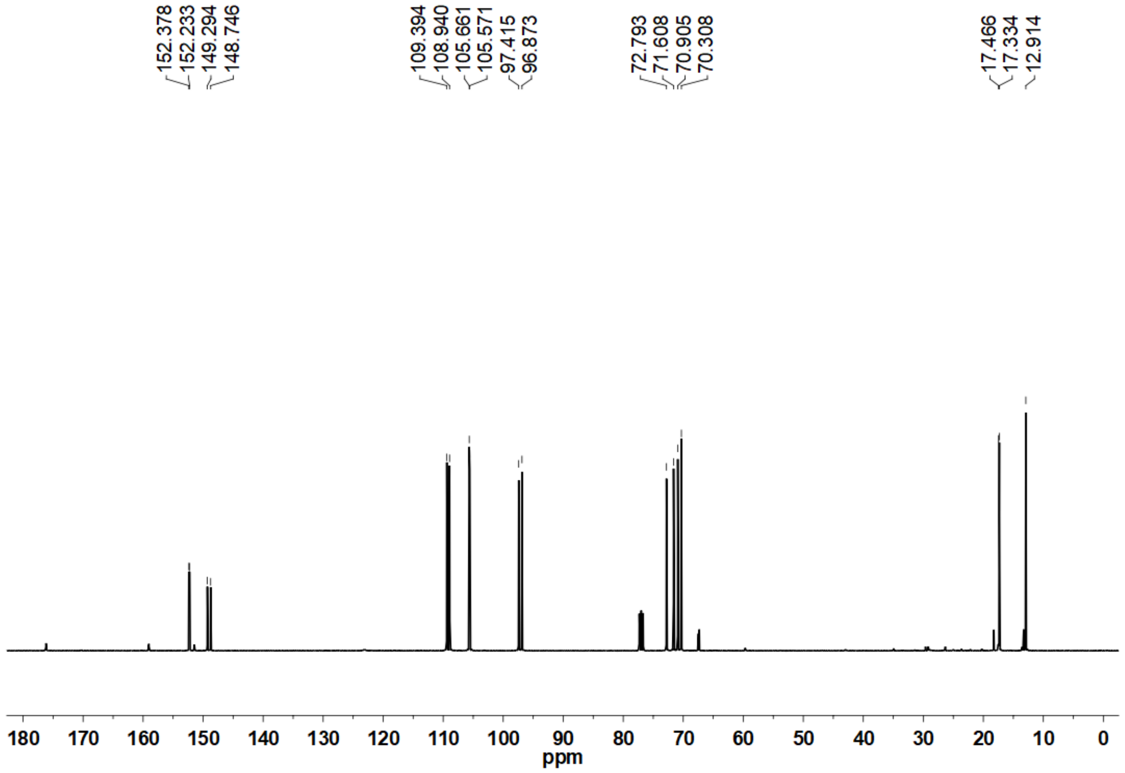

Figure S15. The ^{13}C-NMR spectrum of **5**.

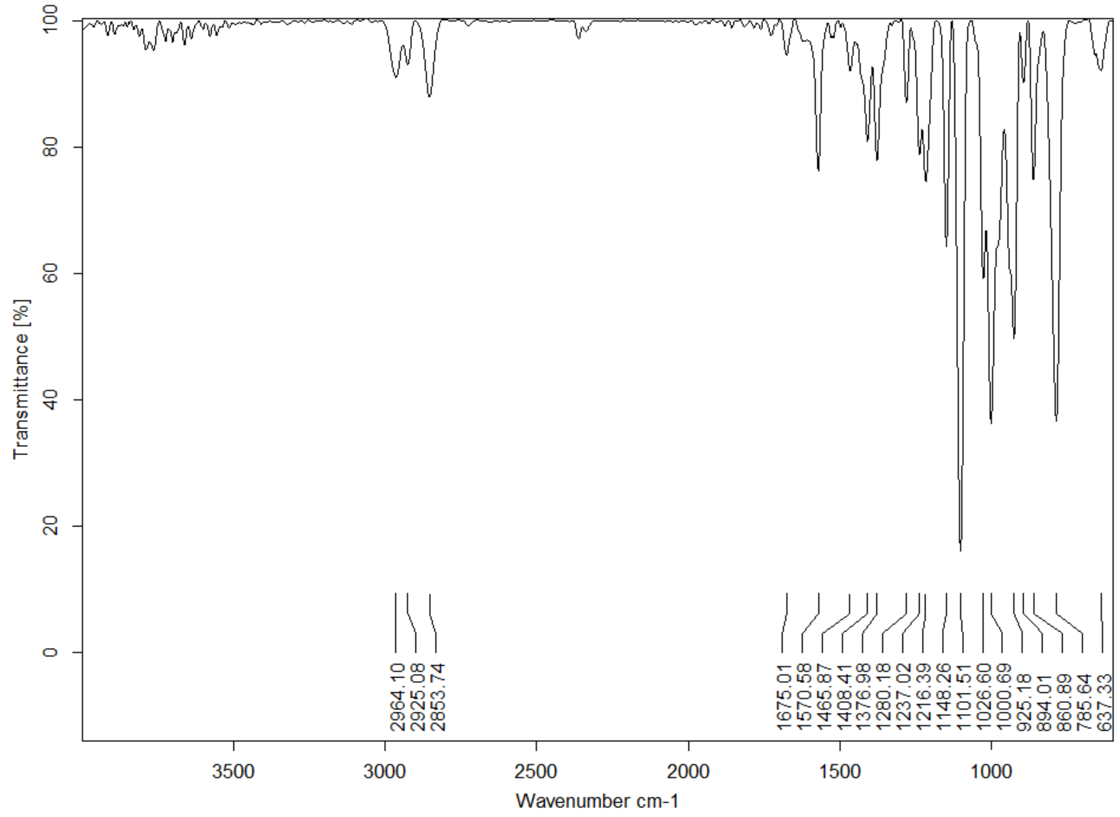

Figure S16. The FTIR spectrum of **6.**

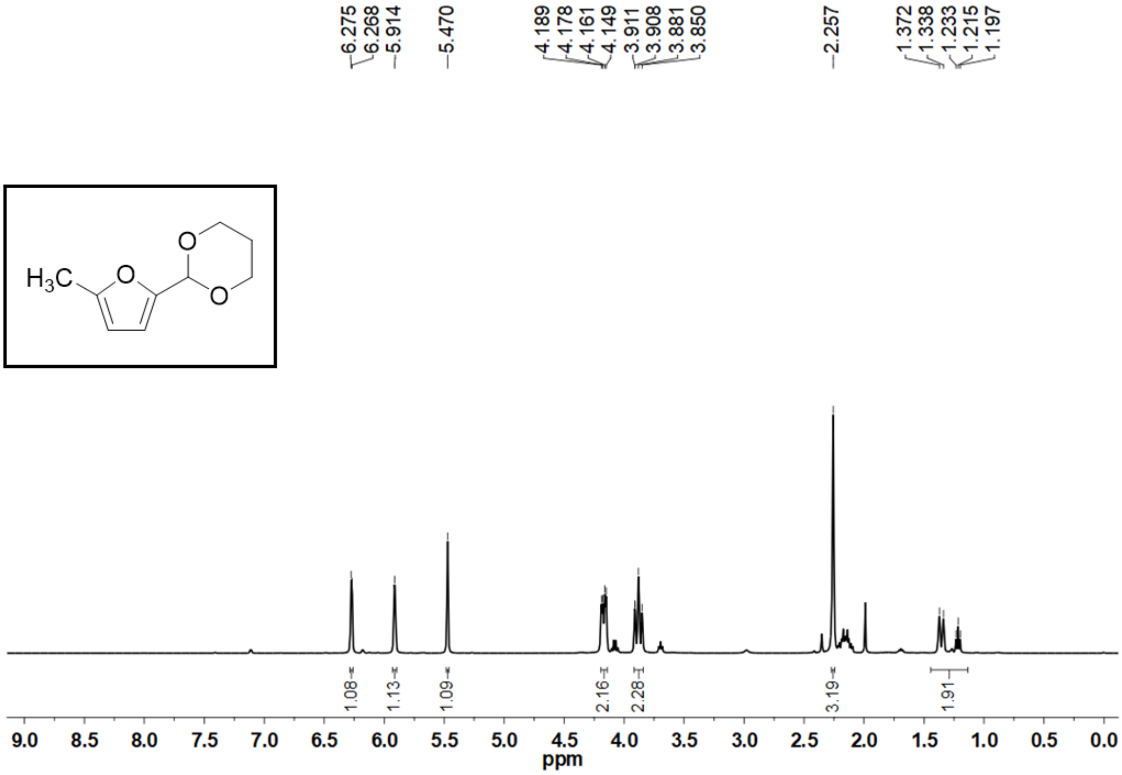

Figure S17. The ^{1}H-NMR spectrum of **6**.

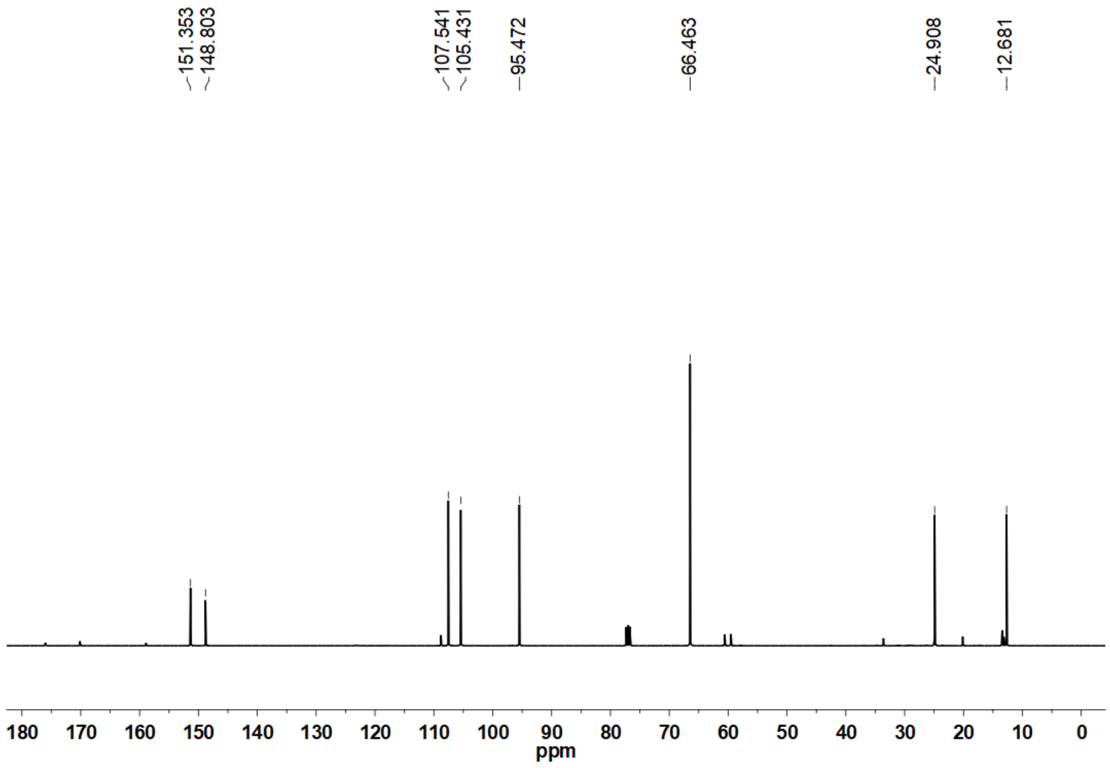

Figure S18. The ^{13}C-NMR spectrum of **6**.

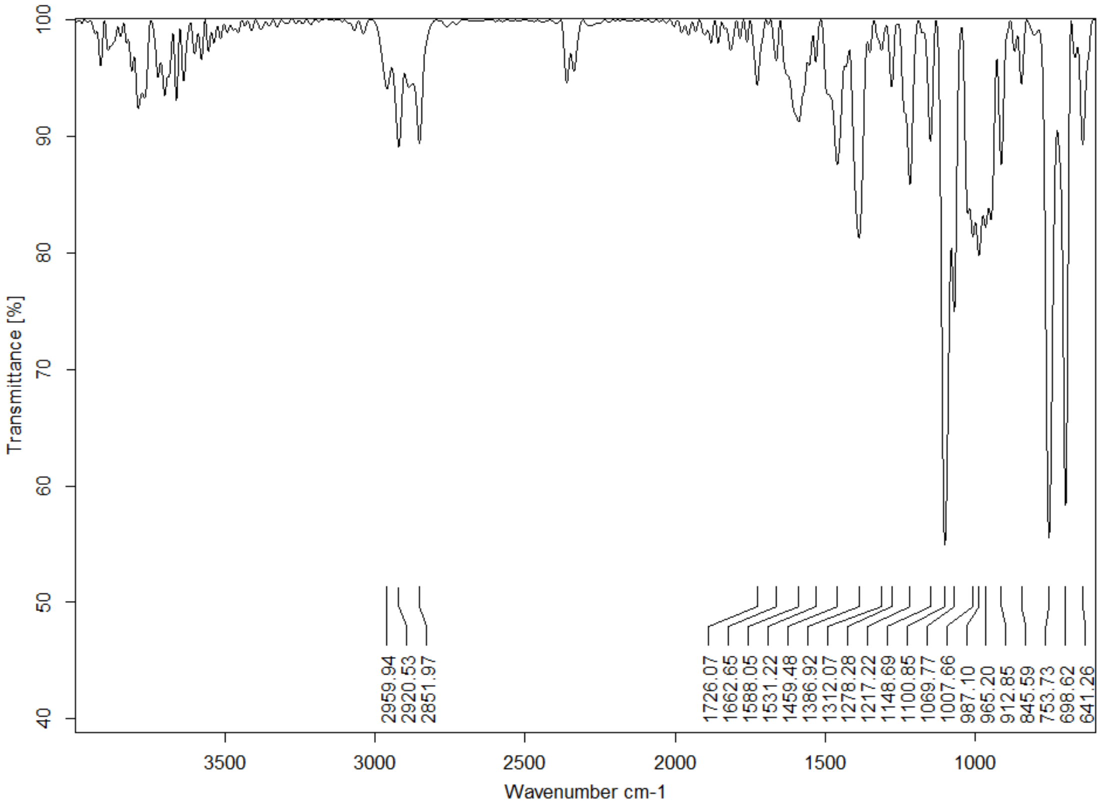

Figure S19. The FTIR spectrum of **7.**

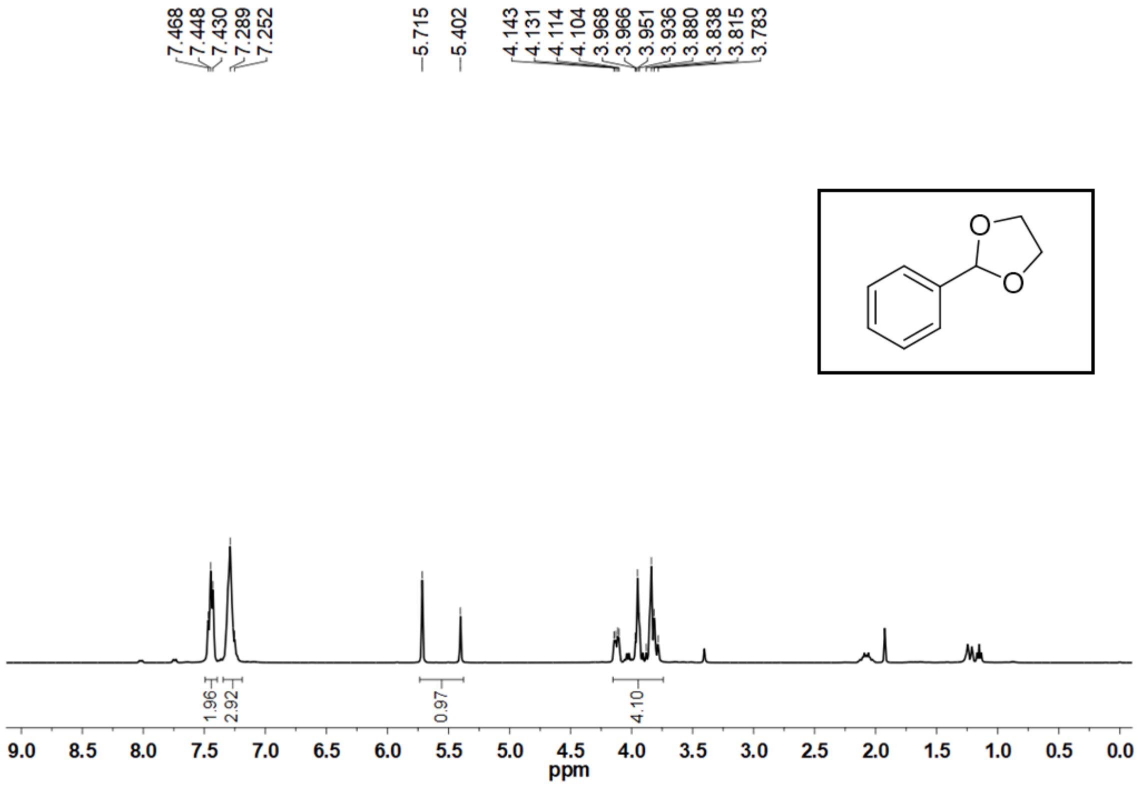

Figure S20. The ^{1}H-NMR spectrum of **7**.

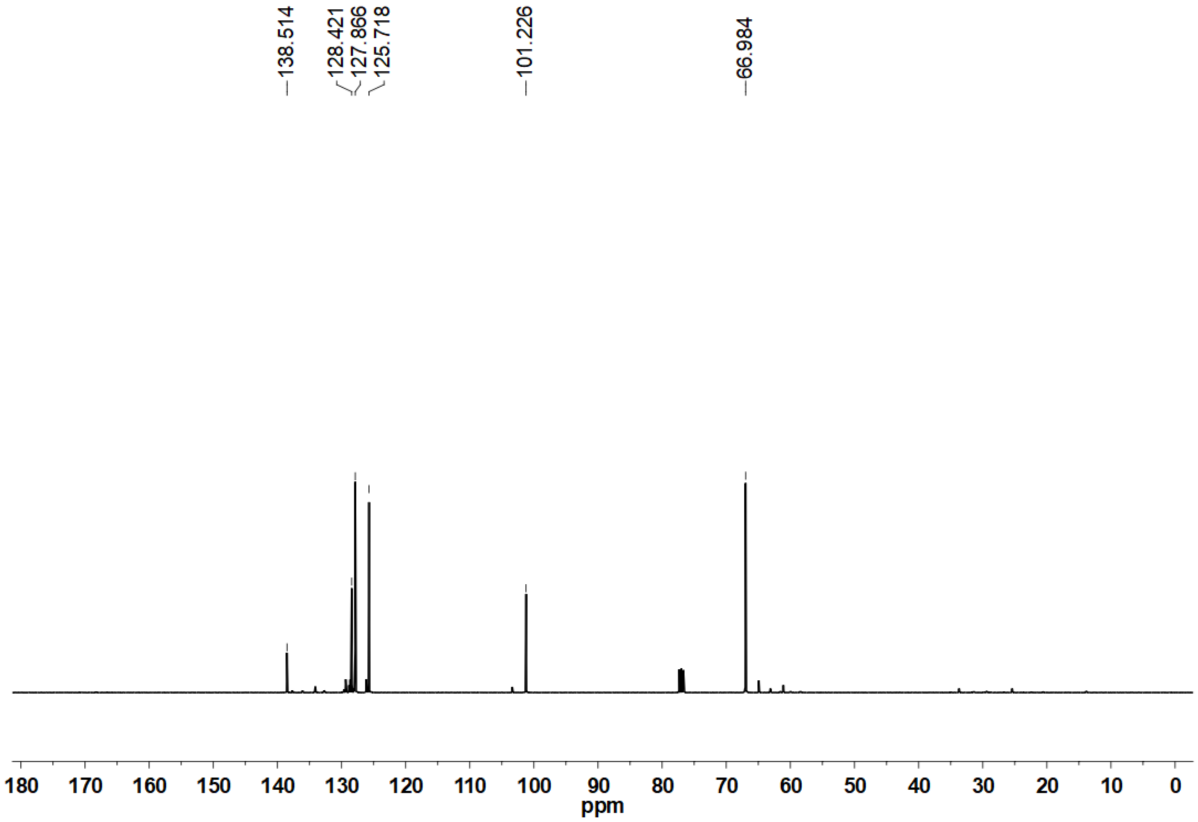

Figure S21. The ^{13}C-NMR spectrum of **7**.

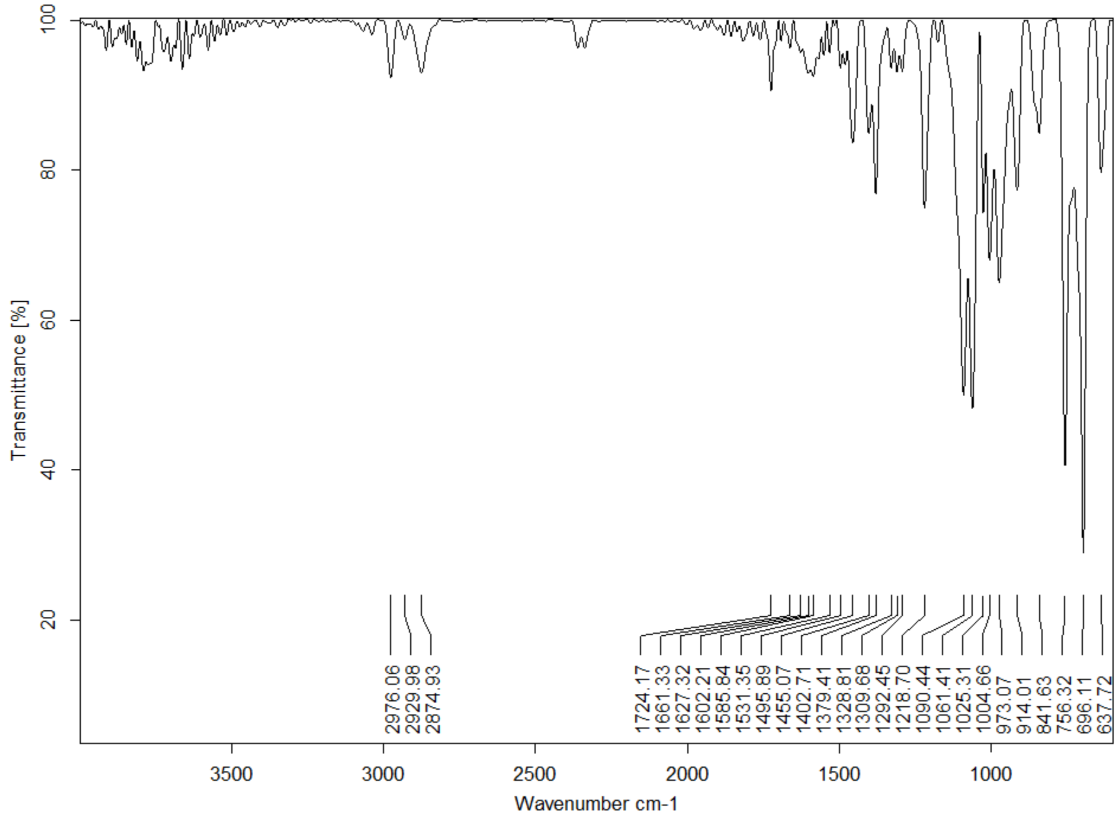

Figure S22. The FTIR spectrum of **8.**

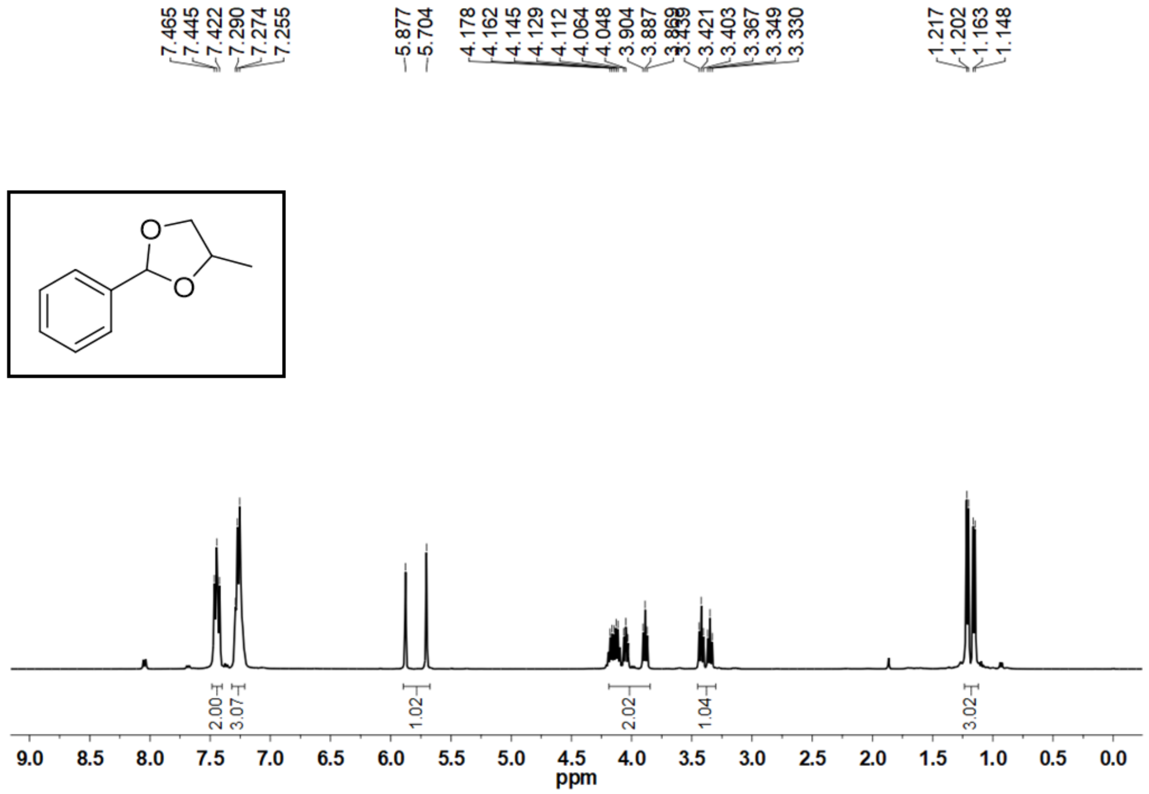

Figure S23. The ^{1}H-NMR spectrum of **8**.

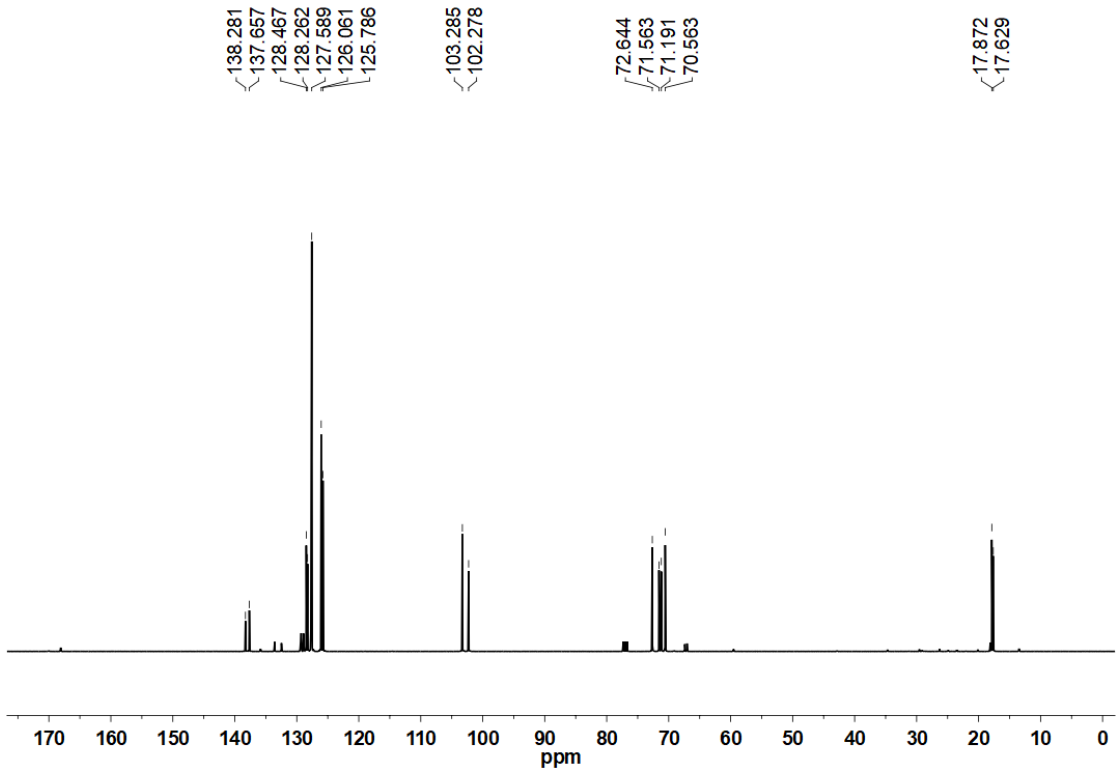

Figure S24. The ^{13}C-NMR spectrum of **8**.

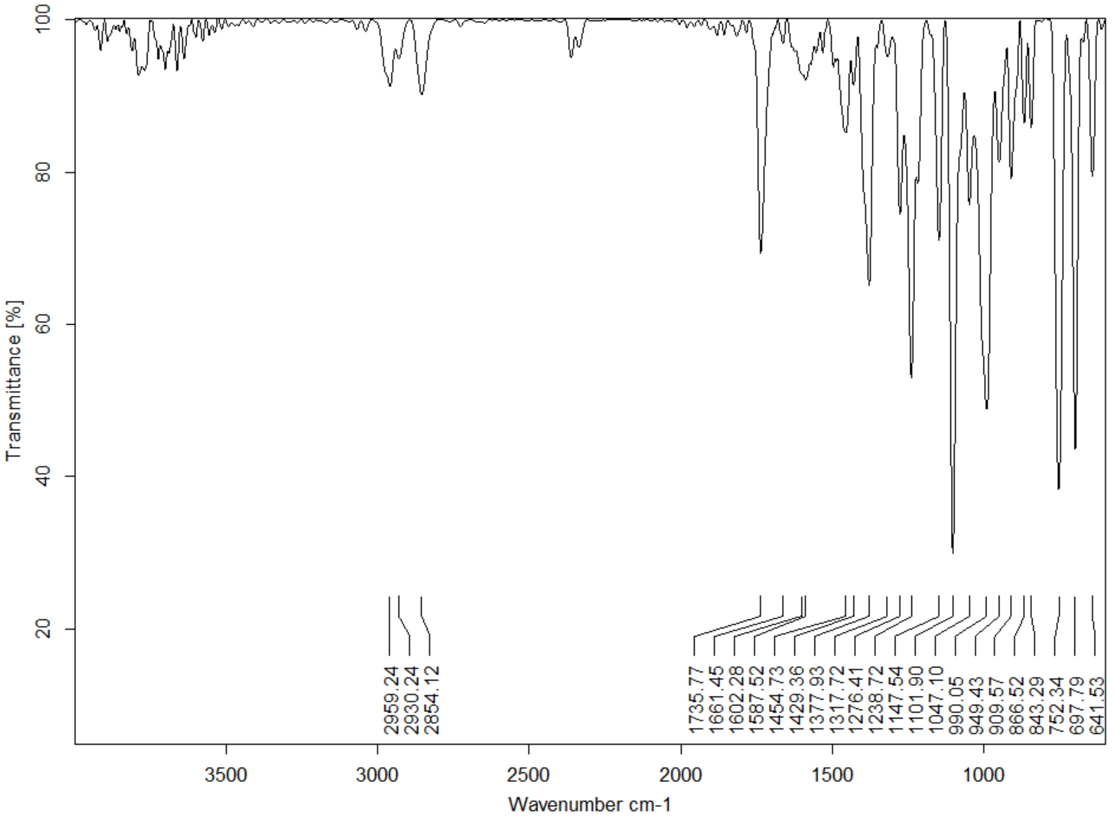

Figure S25. The FTIR spectrum of **9.**

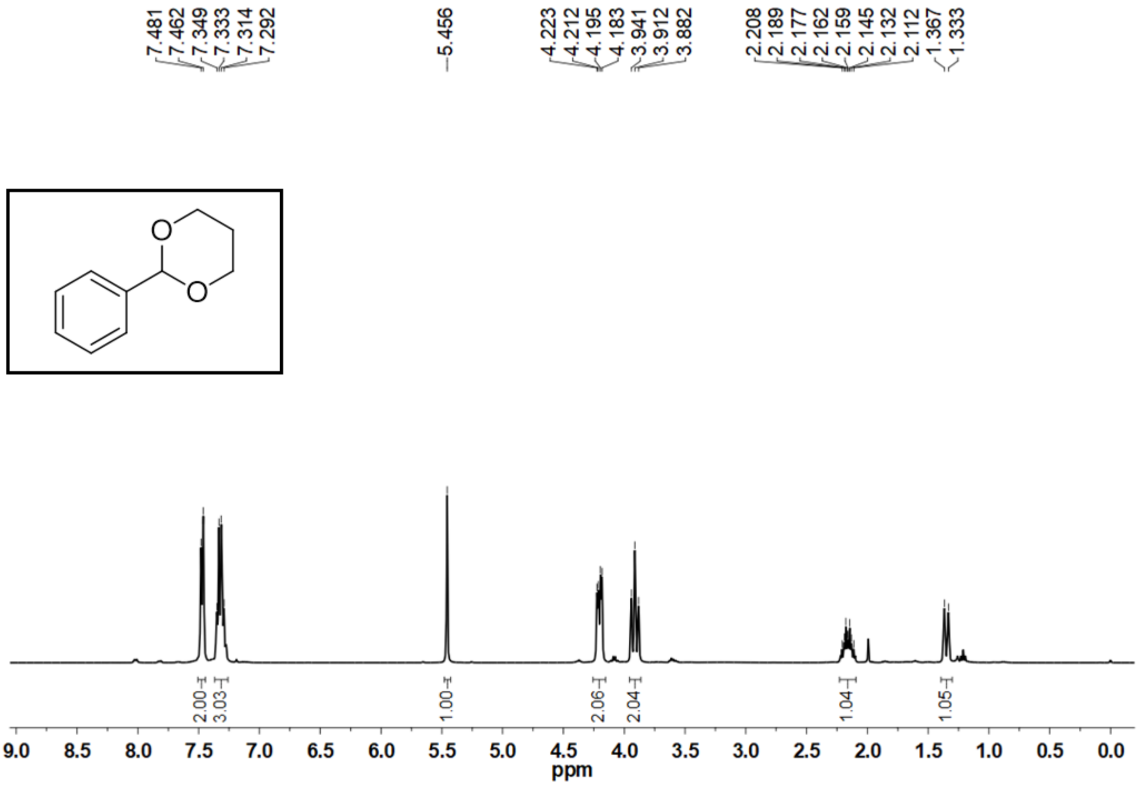

Figure S26. The ^{1}H-NMR spectrum of **9**.

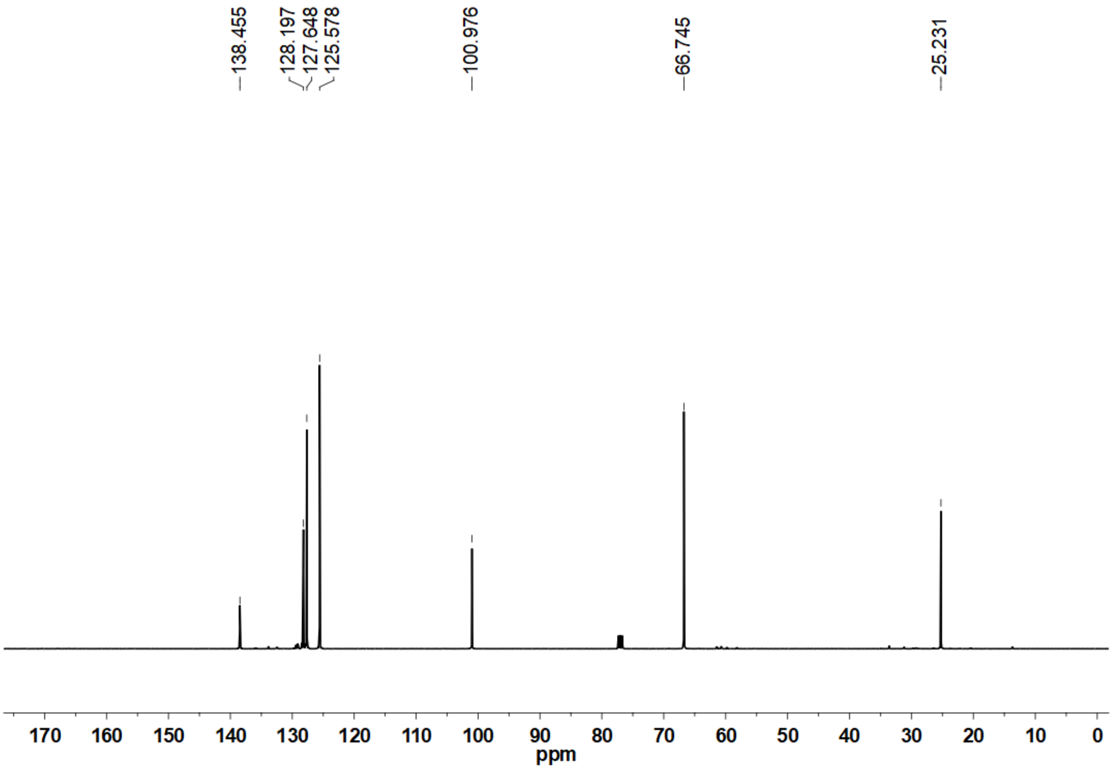

Figure S27. The ^{13}C-NMR spectrum of **9**.

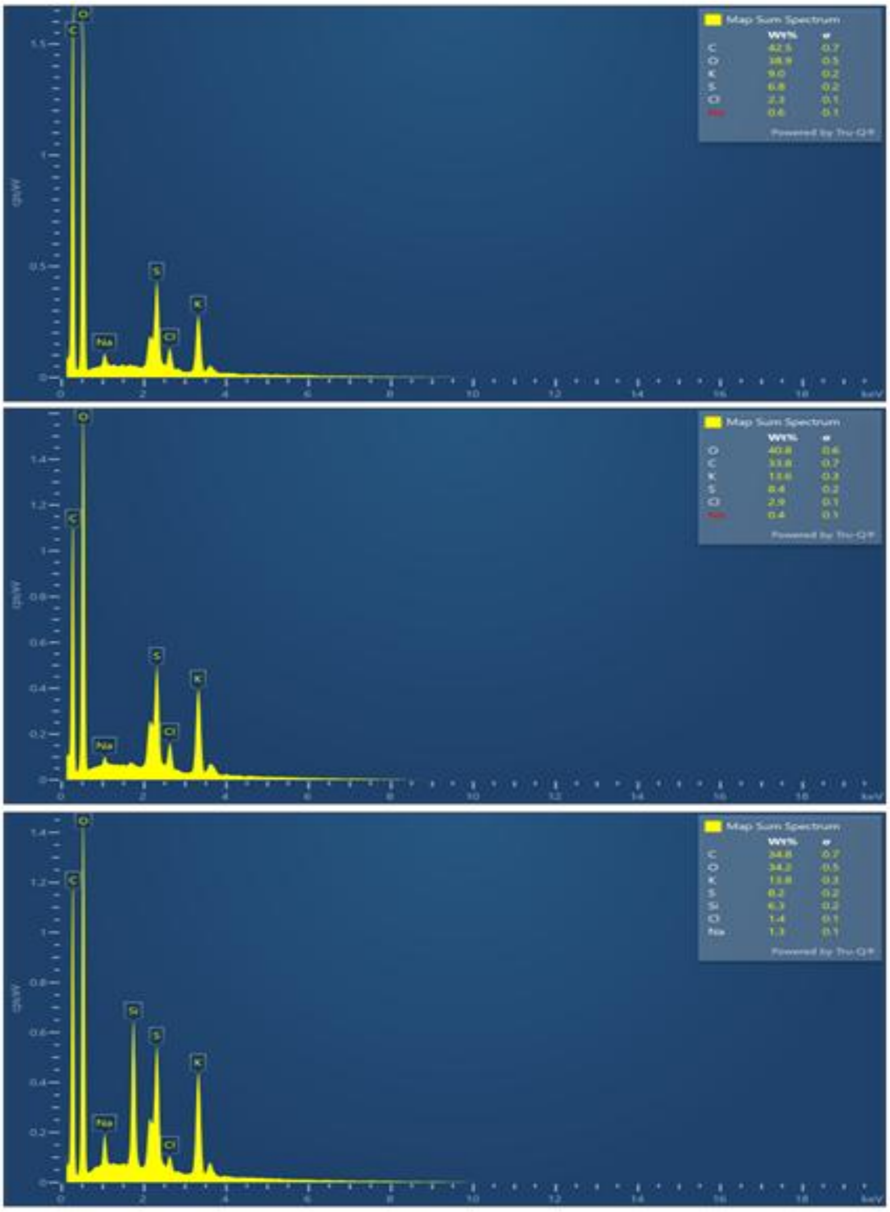

Figure S28. EDS mapping of kC, kCH catalyst, and recycled kCH catalyst.

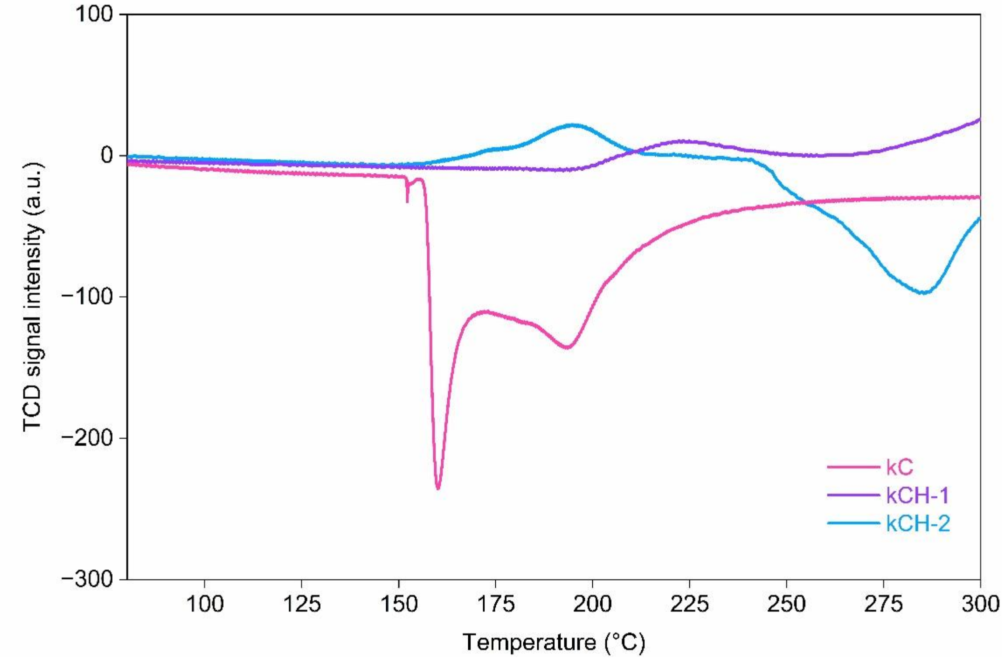

Figure S29. NH3-TPD analysis of kC, kCH-1, kCH-2.

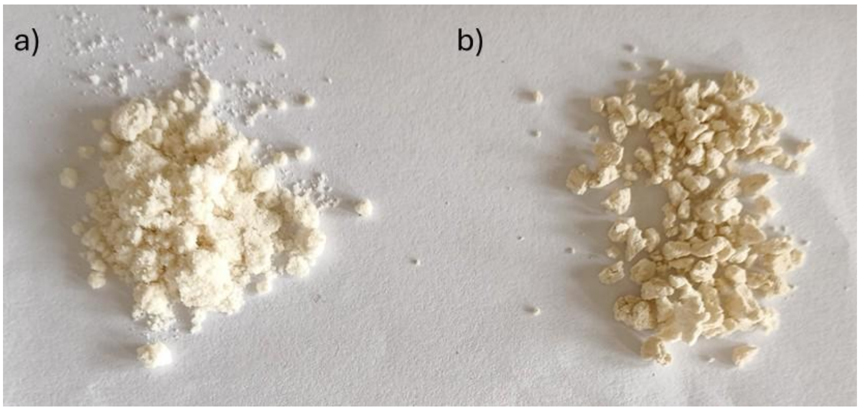

Figure S30. a) Photographic images of the kCH-1 catalyst and b) kCH-2 catalyst.

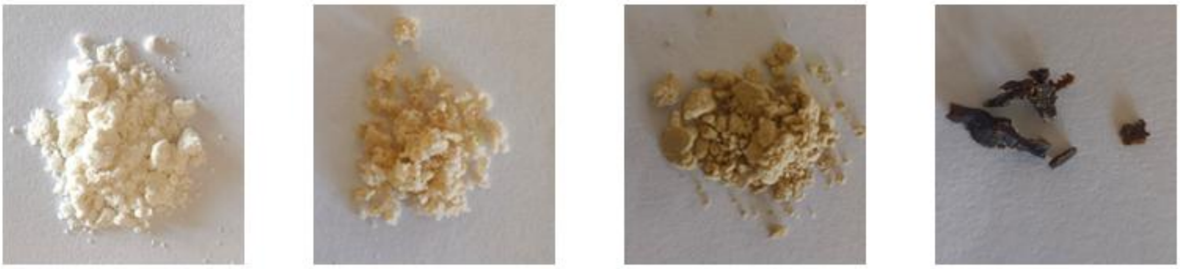

1st cycle 2nd cycle 3rd cycle 4th cycle

Figure S31. Photographic images of the kCH-1 catalyst isolated from the reaction mixture after each catalytic cycle.

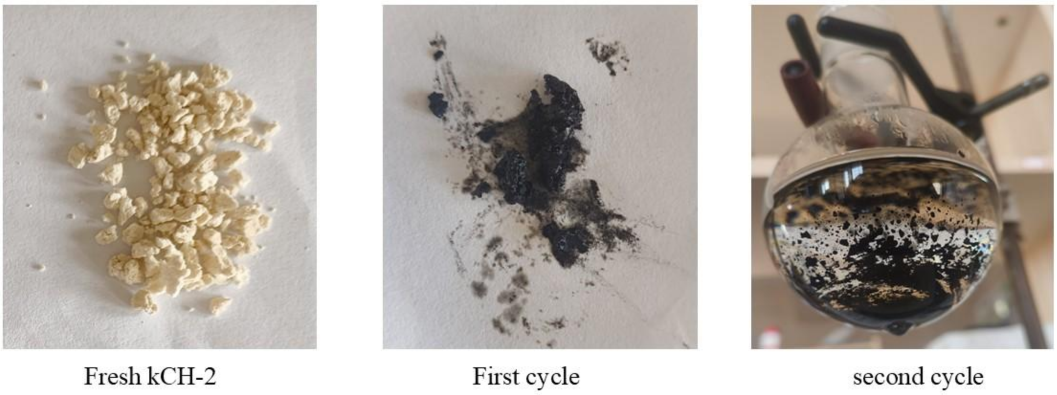

Figure S32. Photographic images of the kCH-2 catalyst isolated from the reaction mixture after each catalytic cycle.

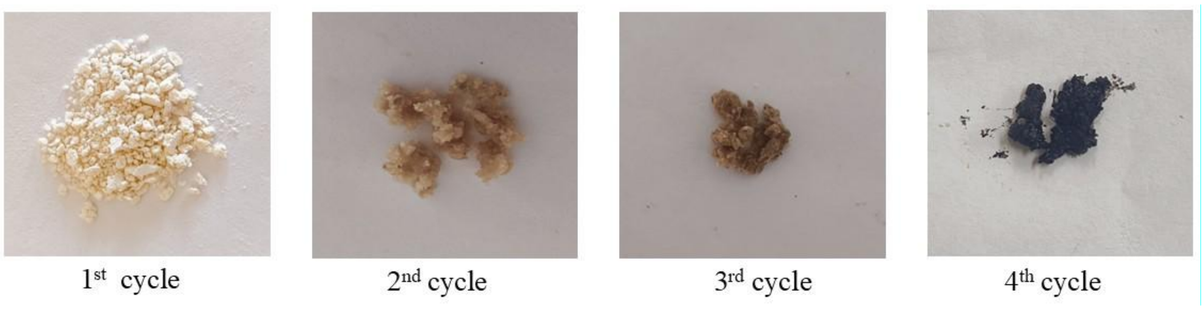

Figure S33. Photographic images of the altered kCH-2 catalyst with KCl isolated from the reaction mixture after each catalytic cycle.

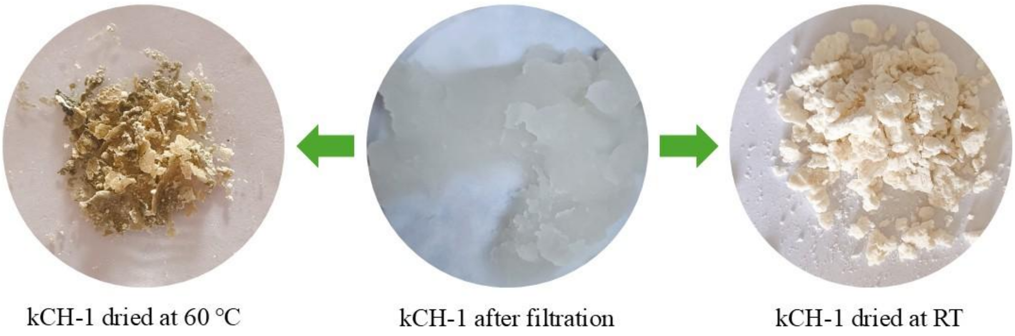

Figure S34. Photographic images of the kCH-1 catalyst dried at 60 °C and RT.

Visual pH indicator test for protonated κ-Carrageenan (kCH)

The color difference observed on the pH indicator strips provides clear visual evidence of the strong Brønsted acidity of protonated κ-carrageenan (kCH). The pH strip was dipped into a suspension of κ-carrageenan (left) that shows mildly acidic or nearly neutral behavior. When the strip was dipped into an aqueous suspension of kCH, it turned to red, suggesting strong acidity (right). The colors of the multicolor pH strip at different pH values are displayed for comparison.

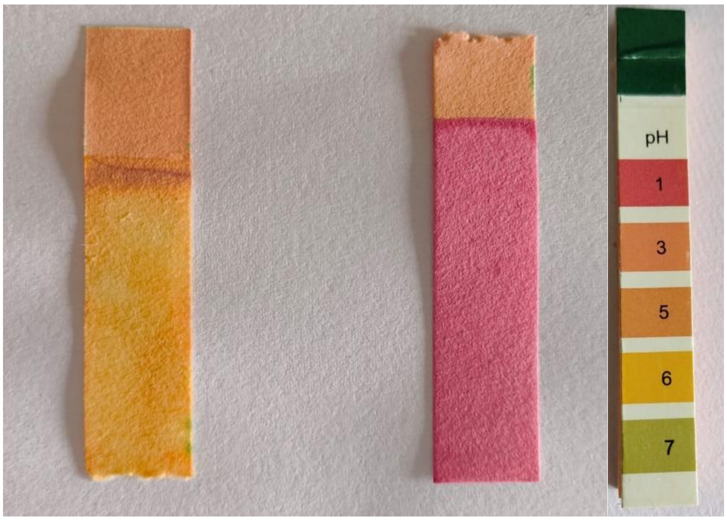

Figure S35. Comparison of the multicolor pH strip using aqueous suspension of kC (left) and kCH catalyst (right).

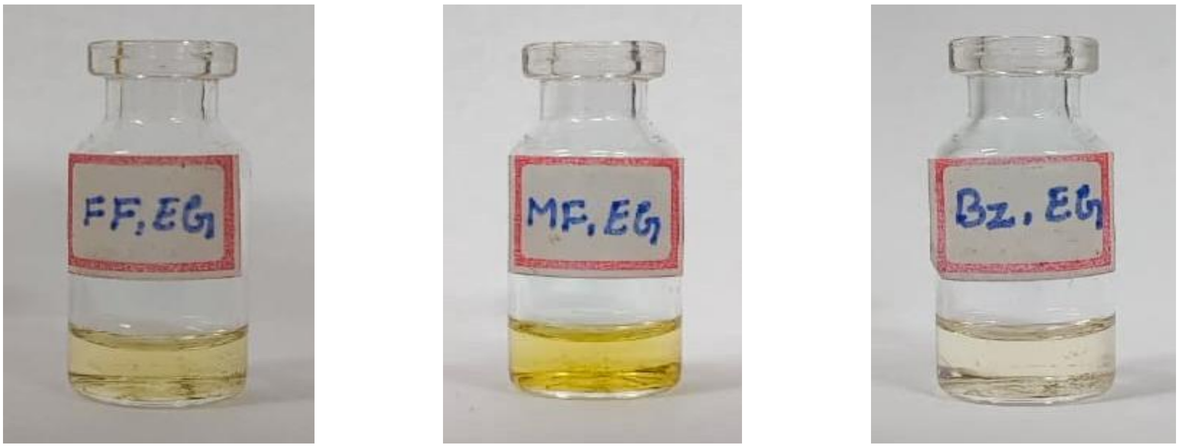

Figure S36. Photographic images of the acetal product: **1** (left), **6** (middle), **9** (right).

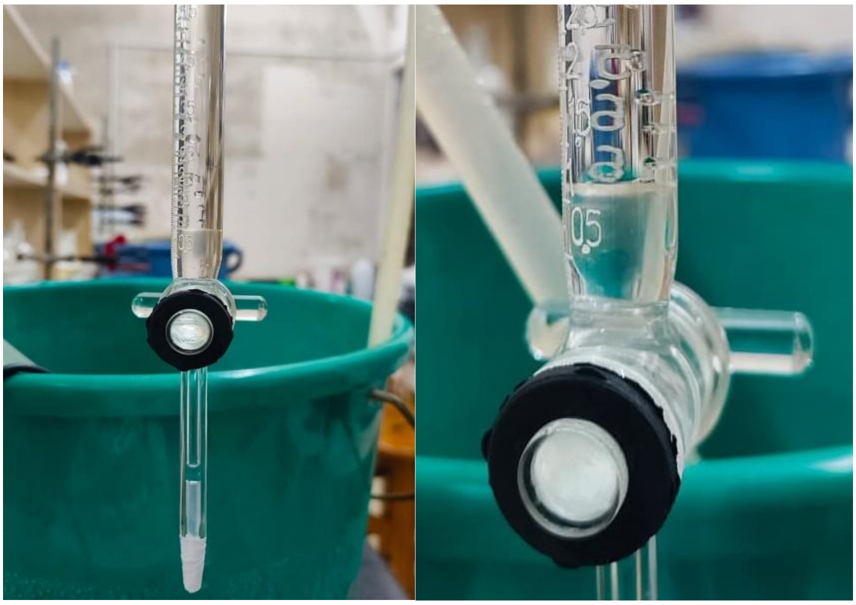

Figure S37. Photographic image of the separated water by azeotropic distillation of cyclohexane in the Dean-Stark apparatus.

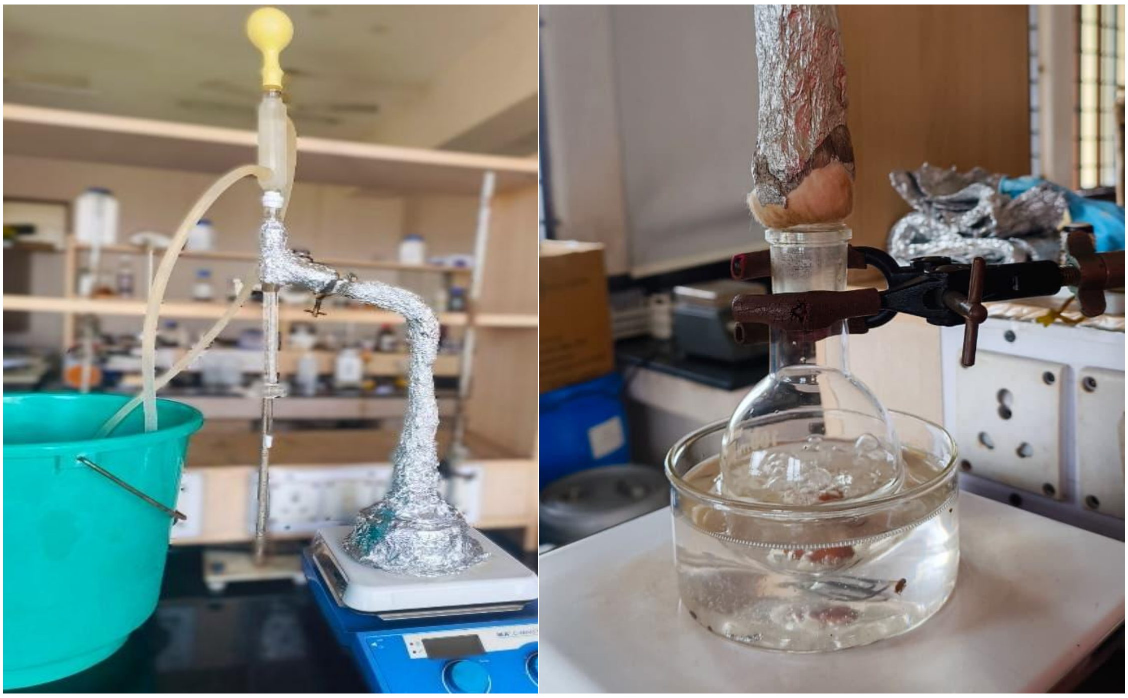

Figure S38. Photographic images of the experimental setup for the acetalization reaction in a Dean-Stark apparatus.

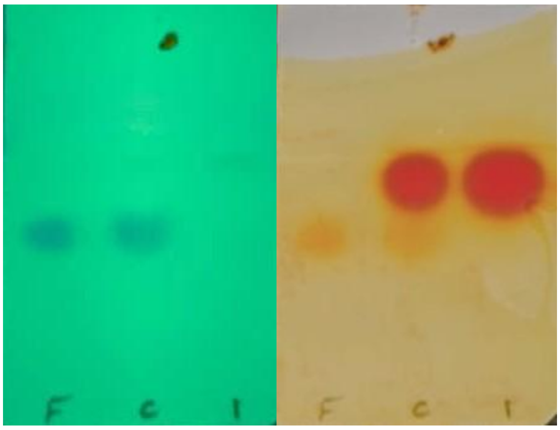

Figure S39. Monitoring the reaction progress and visualization of the acetal **1** by thin-layered chromatography (TLC) eluted with chloroform (F=furfural (R_f=0.65), C=co-spot, P=product (R_f=0.75)). Visualization of **1** in 254 nm UV (left) and acidic 2,4-dinitrophenylhydrazine (DNP) solution.

References

(1) Patil, A.; Shinde, S.; Kamble, S.; Rode, C. V. Two-Step Sequence of Acetalization and Hydrogenation for Synthesis of Diesel Fuel Additives from Furfural and Diols. *Energy Fuels* **2019**, *33* (8), 7466–7472. https://doi.org/10.1021/acs.energyfuels.9b01640.
